# Yeast PAF1 complex counters the pol III accumulation and replication stress on the tRNA genes

**DOI:** 10.1038/s41598-019-49316-5

**Published:** 2019-09-09

**Authors:** Pratibha Bhalla, Ashutosh Shukla, Dipti Vinayak Vernekar, Aneeshkumar Gopalakrishnan Arimbasseri, Kuljeet Singh Sandhu, Purnima Bhargava

**Affiliations:** 1 Centre for Cellular and Molecular Biology (Council of Scientific and Industrial Research), Hyderabad, India; 2 Molecular Genetics Laboratory, National Institute of Immunology, New Delhi, India; 3 Department of Biological Sciences, Indian Institute of Science Education and Research (IISER) – Mohali, Manauli, India; 4 Present Address: UT Southwestern Medical Center, Dallas, Texas USA; 5 Present Address: Institut Curie, Paris, France

**Keywords:** Transcription factors, Genome-wide association studies, Stalled forks, Non-coding RNAs, Transcription

## Abstract

The RNA polymerase (pol) III transcribes mostly short, house-keeping genes, which produce stable, non-coding RNAs. The tRNAs genes, highly transcribed by pol III *in vivo* are known replication fork barriers. One of the transcription factors, the PAF1C (RNA polymerase II associated factor 1 complex) is reported to associate with pol I and pol II and influence their transcription. We found low level PAF1C occupancy on the yeast pol III-transcribed genes, which is not correlated with nucleosome positions, pol III occupancy and transcription. PAF1C interacts with the pol III transcription complex and causes pol III loss from the genes under replication stress. Genotoxin exposure causes pol III but not Paf1 loss from the genes. In comparison, Paf1 deletion leads to increased occupancy of pol III, γ-H2A and DNA pol2 in gene-specific manner. Paf1 restricts the accumulation of pol III by influencing the pol III pause on the genes, which reduces the pol III barrier to the replication fork progression.

## Introduction

Eukaryotic transcription is accomplished by three multi-subunit RNA polymerase (pol) enzymes; pol I, pol II and pol III. Apart from the enzyme itself, the transcription complex (TC) of pol III consists of the basal factors TFIIIC, TFIIIB and TFIIIA- the 5S-specific assembly factor^1,2^. The binding of TFIIIC to the intra-genic promoter elements is required to recruit TFIIIB at the −30 bp position, which in turn recruits pol III^3^. Pol III transcribes small, housekeeping genes of generally 100 bp average length which include the genes for non-coding RNAs like tRNA, 5SrRNA, U6snRNA and others^4^. Pol III transcription is repressed under a variety of stress conditions including nutrient starvation^5–7^. High transcription activity or stalling of the RNA polymerases *in vivo* is documented to generally interfere with the replication fork progression^8^. The tRNA genes are highly transcribed *in vivo*^9^, which causes the replication on them to slow down^10,11^. The resultant fork pausing is reported to make them prone to the replication stress-induced DNA damage *in vivo*^10–12^. Loss of the barrier to replication-fork passage due to a mutation in the TFIIIC or TFIIIB binding site on tRNA genes resulting in reduced replication-fork stalling^10,11^, suggests a strong link between the transcription and replication on the tRNA genes. The fork pausing is reported to reduce on the tRNA genes in caloric-restricted cells^13^.

One of the transcription factors of pol II, the Paf1 complex^14^ associates with pol II^15^ and has two main activities on pol II-transcribed genes^16,17^. It is required for transcription elongation by pol II and pol I^18–20^ and for coordinating the co-transcriptional histone modifications on the gene body nucleosomes^15,21^. However, its role in proper 3′-end formation of the transcripts^22^ and several other non-transcriptional processess like DNA repair, cell cycle, gene silencing etc^16,23^. are not well understood yet. Paf1 complex (PAF1C) is composed of five subunits Paf1, Rtf1, Leo1, Cdc73 and Ctr9^19,24^. Cells lacking different Paf1 complex subunits show different genotoxin sensitivities^25,26^. Several studies in the past have found that despite being subunits of the same complex, Rtf1 does not behave similar to other subunits in yeast^19,25–29^. The Paf1 (and not Rtf1) deletion affects protein levels of other PAF1C subunits as well^30^. This suggests that the *PAF1* deletion effects may be more severe than the *RTF1* deletion effects. In our earlier efforts to find the possible regulatory partners of the pol III TC, we had found the Paf1 complex in our proteomic experiments^27^.

Phosphorylation of the Ser139 of mammalian histone H2A.X (and Ser129 residue of yeast H2A) generates γ-H2A.X (yeast γ-H2A), the marker of the DNA damage^31,32^. As expected, we found low levels of γ-H2A and the DNA pol2 subunit of the pol ε on the yeast pol III-transcribed genes, which increased upon exposure to the genotoxin HU or Paf1 deletion. Pol III levels on the genes reduced upon genotoxin exposure while Paf1 influenced the effect of the genotoxin HU but not MMS. We found Paf1 associates with the pol III TC and shows gene-specific, low occupancy levels on the pol III-transcribed genes. It does not affect the low level nucleosomes near the pol III-transcribed genes, which carry very low levels of active state H3K4 and H3K36 histone methylation marks. It restricts pol III occupancy levels on the genes, as pol III levels increase several fold on all the tested genes in the *paf1∆* cells. This pol III accumulation represents its stalling as the mature tRNA levels show only a small increase in the *paf1∆* cells. These results suggest an inhibitory influence of Paf1 on pol III-transcribed genes, which may serve to protect these highly transcribed, known replication fork pause sites^10,11^ against the DNA damage.

## Results

### Paf1 complex associates with the pol III-transcribed genes in a gene-specific manner

**Figure 1:**
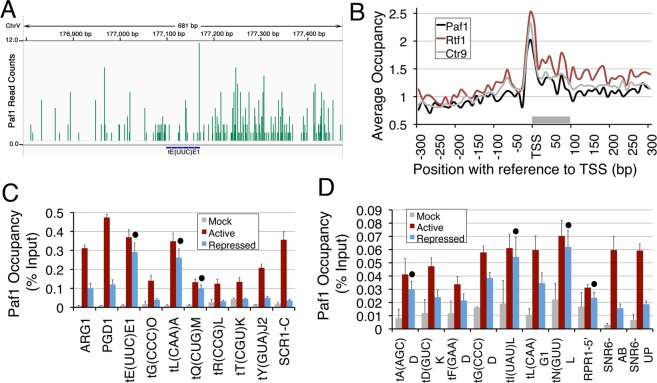
Paf1 localizes to the pol III-transcribed genes. Low level of Paf1 occupancy is found at the pol III-transcribed gene loci. The genome-wide data on Paf1 occupancy^33^ was analysed. (**A**) Screenshot of a view with Integrative Genomic Viewer (IGV) of Paf1 occupancy on the gene *tE(UUC)E1* in a window spanning 300 bp upstream and downstream of the gene ends. (**B**) Similar average occupancy profiles were found for the PAF1C subunits on the tRNA genes. The average occupancies on both 300 bp upstream and downstream of TSS are plotted. The grey bar marks the tRNA gene region on the X-axis. (**C**,**D**) Paf1 occupancy over pol III-transcribed genes relative to TelVIR region were estimated by ChIP and Real time PCR in active and repressed states. The changes were found to be significant (p value < 0.04) for all except those marked with a dot.

In order to ascertain the PAF1C presence on the pol III-transcribed genes *in vivo*; we analysed two sets of the earlier published and available genome-wide occupancy data^33,34^. Our analysis of the earlier ChIP-exo, genome-wide occupancy data^33^ showed that as compared to pol II-transcribed genes, the occupancy levels of Paf1 were lower on the pol III-transcribed genes (Fig. 1A,B and Supplemantary Fig. S1). Further analysis of this data^33^ found highly similar occupancy profiles of all five subunits of the Paf1 complex (Fig. 1B and Supplemantary Fig. S2A) on the tRNA genes. The average occupancy profiles of all five PAF1C subunits show lower levels on the gene body and a sharp peak (Fig. 1B and Supplemantary Fig. S2A) at the −10 bp position between the box A at +20 bp position and the binding site of the pol III transcription initiation factor TFIIIB at the −30 bp position^35^. As subunits of the same complex, Ctr9 occupancy on the individual genes (Supplementary Fig. S2B) showed a positive correlation coefficient with both Paf1 and Rtf1 occupancies (r = 0.414 and 0.471 respectively).

Similar to Paf1 (Supplementary Fig. S1), we noticed wide variations in levels of Rtf1^34^ on the individual pol III-transcribed genes (Supplementary Fig. S2C). Many of the tRNA genes are found in the intergenic regions, in close proximity of the pol II-transcribed gene-ends. Re-analysis of the data after filtering out the tRNA genes with pol II-transcribed gene ends within 300 bp on their both the sides showed changes only in the downstream but not on the tRNA gene region in the average Rtf1 occupancy profiles (Supplementary Fig. S2D). Therefore, the observed occupancy on the tRNA genes is not due to the vicinity of pol II-transcribed genes.

Both Paf1 (Supplementary Fig. S1) and Rtf1 (Supplementary Fig. S2C) levels show gene-specific variations on the individual pol III-transcribed genes. Using pol II-transcribed *ARG1* and *PGD1* genes as positive controls, we checked for the presence of Paf1 on several pol III-transcribed genes by the ChIP-Real Time PCR assay (Fig. 1C,D). We found Paf1 on all of the tested tRNA genes, which could be grouped into comparatively higher (Fig. 1C) or lower (Fig. 1D) occupancy level genes. As compared to the *ARG1* gene, Paf1 level was ~2–3 fold lower on the high (Fig. 1C) and >3 fold lower on the low occupancy group genes (Fig. 1D). The non-tRNA genes *SNR6*, *RPR1* show low Paf1 occupancy (Fig. 1D) while *SCR1* (Fig. 1C) could be grouped with higher occupancy genes (amplicons shown in the Supplementary Fig. S2E). The interaction of Paf1 with the pol III TC in both active and repressed states and the presence of all five subunits in the pol III TC interactome^27^ and on the pol III-transcribed genes (Fig. 1) affirm the interaction of pol III TC with the complete Paf1 complex.

Interestingly, under repression, ten of the total seventeen tested pol III-transcribed genes show Paf1 loss while rest of the genes do not show any significant change. The gene-specific occupancy levels and their changes under repression suggest that Paf1 may have gene-specific positive or negative influence on the pol III-transcribed genes, similar to earlier reports on pol II-transcribed genes^14,36^. These results indicate that the Paf1 association may be linked to the active or repressed state of the genes and not to a particular step of the transcription process.

### Paf1 does not affect nucleosomes near the pol III-transcribed genes

**Figure 2:**
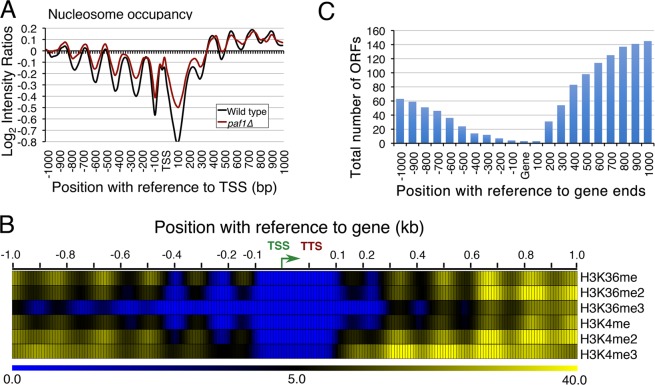
Paf1 does not affect chromatin around tRNA genes. (**A**) Paf1 does not influence nucleosome occupancy^41^ on tRNA genes. (**B**) Methylation levels on K4 and K36 residues of the histone H3^49^ are very low near tRNA genes. Heat map is shown for 1 kb upstream and downstream of the gene. Color gradient code is shown at the bottom, TTS and TSS (bent arrow) are marked. (**C**) ORF content in the 1 kb up- and downstream regions of the tRNA genes is shown. The number of ORFs were counted at every 100 bp upstream or downstream of tRNA gene body using the available annotations in the SGD database (https://www.yeastgenome.org). Only those ORFs found on the same strand as tRNA were counted.

Paf1 is reported to influence nucleosome occupancy on pol II-transcribed gene regions^37–39^. As transcription on pol III-transcribed genes is modulated by the positioned nucleosomes flanking the nucleosome-free region (NFR) housing the gene body^40^ and Paf1 is found within the NFR on the pol III-transcribed loci, we retrieved and analysed the published data on the nucleosome occupancy on these genes in the wild type and *paf1∆* cells^41^. Occupancy of the gene-flanking nucleosomes shows only a small change in the *paf1∆* cells (Fig. 2A). An apparent increase in intensity at the gene body is not related to nucleosome occupancy, as explained later. Therefore, Paf1 does not influence positions or occupancies of gene-flanking nucleosomes on the tRNA genes.

### Low histone methylations on the pol III-transcribed genes

Paf1 is known to mediate repression of a pol II-transcribed gene *ARG1* by preventing recruitment of the histone acetyltransferase Gcn4 to the *ARG1* promoter and subsequent histone H3 acetylation^42^. The Paf1 complex is required for recruitment of Set1 and Set2 methyltransferases on pol II-transcribed gene bodies during elongation^17,43^, which mark the active genes with H3K4 and H3K36 methylations, respectively^44,45^. Unlike the earlier reported presence of active chromatin marks near human pol III-transcribed genes^46–48^, our analysis of the published genome-wide data on histone modifications^49^ in yeast revealed absence/extremely low levels of H3K4 and H3K36 methylations, upstream of TSS on the tRNA genes (Fig. 2B). As compared to the 5′ upstream regions, we noticed a larger propensity of the pol II-transcribed genes towards 3′ ends of the tRNA genes (Fig. 2C). This suggests that while the higher than upstream methylation levels may be related to Paf1-occupied pol II-transcribed genes in the downstream region (Fig. 2B); on tRNA genes, Paf1 may not have a role in histone methylations.

As compared to the genome-wide levels, nucleosome occupancy around tRNA genes is known to be low^50,51^. Therefore, low levels of H3K4 and H3K36 methylations found on pol III-transcribed tRNA genes may be due to low nucleosome occupancy and/or low Paf1 levels on these gene regions. Further validations and measurements of these methylations in *paf1∆* cells will be required to get a better understanding of Paf1 role in histone methylations on different pol III-transcribed genes.

### Paf1 has a gene-specific influence on the transcription of tRNA genes

**Figure 3:**
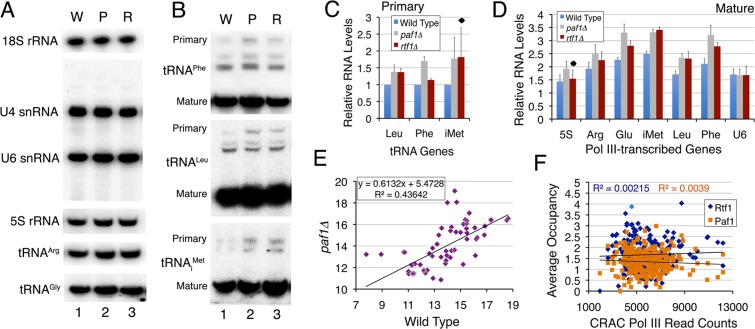
Paf1 has repressive role in RNA pol III transcription. (**A**,**B**) Estimation of transcript levels in *paf1Δ* and *rtf1∆* cells by northern analysis for different genes in wild type [W], *paf1Δ* [P] and *rtf1∆* [R] cells. Radiolabeled oligonucleotide probes against 18 S rRNA (pol I transcribed), U4 snRNA (pol II transcribed) and pol III transcribed U6 snRNA, 5 S rRNA and different tRNA genes were used individually. Primary and mature transcript level positions are marked. (**C**) Quantifications of pre-tRNA products in panel B for tRNA_i_^Met^, tRNA^Phe^ and tRNA^Leu^ normalized with U4 levels. Averages and scatter from three independent experiments are plotted. (**D**) Quantifications of mature RNA products in panel A for 5 S rRNA and tRNA_i_^Met^, tRNA^Arg^, tRNA^Glu^, tRNA^Phe^ and tRNA^Leu^ normalized with U4 levels. Average and scatter from three independent experiments are plotted. Dots mark the statistically insignificant changes. (**E**) Paf1 deletion causes gene-specific differences in the individual tRNA levels between the wild type and mutant cells. The log_2_ transformed, normalized read counts of each tRNA from the wild type and *paf1Δ* cells obtained in the HySeq data are compared. (**F**) Paf1 and Rtf1 mean occupancies (^33^, between −205 to + 155 bp) are not correlated to the pol III levels associated with the nascent RNAs estimated by the CRAC (UV Crosslinking and Analysis of cDNA) method^53^.

Paf1 has been shown to promote transcription of pol II-transcribed genes through chromatin *in vitro*^20^, whereas genome-wide studies have reported an unequal effect of Paf1 deletion on expression of the Paf1-associated pol II-transcribed genes^14,36^, suggesting a role for it in transcription of only a limited number of genes. Paf1 is reported to have a repressive role in the expression of pol II-transcribed *SER3* and *ARG1* genes^38,42^. Our measurements of transcription in wild type and Paf1 or Rtf1 deletion cells by northern probing did not find any change in levels of 18S rRNA (pol I transcript), U4 (pol II transcript) and pol III transcripts U6 snRNA and 5S rRNA (Figs 3A and S3A, S3B). Since the pre-tRNAs (primary transcripts) are generally difficult to detect due to their low levels as compared to mature tRNA levels, we could measure pre-tRNA levels of only three tested genes by these estimations (Fig. 3B). Nevertheless, as compared to wild type, both primary (p < 0.05) and mature (p < 0.03) tRNA levels showed a small but significant increase for the tested genes in both the deletion strains (Fig. 3C,D). Although measurement of primary transcript from a larger number of genes may be required, above results suggest a repressive role for PAF1C in the pol III transcription. Our measurements by tRNA HySeq method^52^ on total tRNA pools covering a larger number of genes revealed both increase and decrease of relative tRNA levels (Supplementary Fig. S3C). No correlation was found between the tRNA levels in the wild type and *paf1∆* cells (Fig. 3E), which signifies gene-specific differences between them. These changes were found statistically insignificant (Fig. 3E) while no changes were seen in the *rtf1∆* cells (Supplementary Fig. S3D).

Most of the tRNAs are represented by multiple isogenes, which make their estimations by using gene body probes, erroneous. Therefore, pol III occupancy is considered a better measure of the transcription activity of these genes. We did not find a correlation between Rtf1 or Paf1 occupancies with the recently published levels of pol III (Fig. 3F) bound to the presumably primary transcripts of all the tRNA genes^53^. Only a poor correlation (R = 0.1547; Supplementary Fig. S3E) is found between the pol III levels bound to the genomic sites^40^ and to the primary transcripts (CRAC, 53). These may be the reasons that we found only small changes in transcript levels due to *PAF1* or *RTF1* deletions. Nevertheless, in the light of the short transcribed regions and nucleosome-free gene bodies, the significance of the association of Paf1 complex with pol III-transcribed genes may be different from its known roles on the pol II-transcribed genes.

### Paf1 deletion increases DNA damage at the pol III-transcribed genes

**Figure 4:**
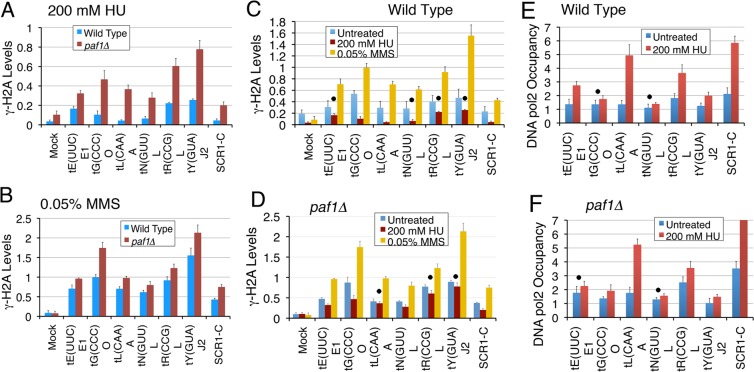
Paf1 is required to counter the replication stress at pol III-transcribed genes. Levels were measured in cells with or without exposure to genotoxins by using ChIP and Real Time PCR method. Dots mark the genes, which do not show significant change under a specific condition (p value > 0.05). Rest of the changes are significant. **(A)** and **(B)** As compared to the wild type, γ-H2A levels increase in *paf1Δ* cells when both types of cells are similarly treated with (**A**) HU; all p values < 0.0045 or (**B**) MMS; all p values < 0.05. (**C**,**D**) As compared to the levels in the untreated condition, the γ-H2A levels increase (p value < 0.009) after MMS exposure. (**C**) As compared to the low, normal (untreated) levels in the wild type cells, γ-H2A levels decrease when exposed to HU. The p values for the genes marked with a dot vary between 0.06 and 0.076; rest are < 0.035. **(D**) Higher than the wild type γ-H2A levels in the *paf1Δ* cells mostly do not change when exposed to HU. The p values for the genes marked with a dot vary between 0.05 and 0.16; rest are < 0.04. (**E**,**F**) The DNA pol2-9XMyc occupancy on the tRNA genes increases upon exposure to 200 mM HU for 2 hr. (**E**) In the wild type cells, DNA pol2 shows gene-specific increases on most of the genes, except *tG(CCC)O* (p value = 0.061 and *tN(GUU)L* (p value = 0.052), marked with a dot. (**F**) HU treatment of the *paf1Δ* cells takes the DNA pol 2 levels on all the tested genes to same as that in the wild type cells, except on the *SCR1* gene where levels were found beyond the given scale (increase of ~6.8 fold).

Genome-wide mapping of γ-H2A enrichment has been reported to represent the fragile as well as replication fork stalling sites in the genome^12^. Average occupancy profile of Paf1 (Fig. 1B) looks similar to that of γ-H2A^12^ on the tRNA genes, which was found located just upstream to TSS of the tRNA genes^12^. We found low levels of γ-H2A on the pol III-transcribed genes in the wild type cells under normal growth conditions (Supplementary Fig. S4A), which represent the slow replication on these highly transcribed genes. The higher than the wild type levels of γ-H2A at individual genes in the *paf1∆* cells (Supplementary Fig. S4B) suggest gene-specific increase of DNA damage in the *paf1∆* cells. As compared to the wild type cells, higher γ-H2A levels were found in the *paf1∆* cells, when both cell types were similarly exposed to the genotoxins HU (Fig. 4A) or MMS (Fig. 4B). The increase in γ-H2A levels in the *paf1∆* cells was more pronounced under HU (2–8.5 fold, p < 0.005) than under MMS (1.3–1.8 fold, p < 0.05) exposure, varying in a gene-specific manner (Supplementary Fig. S4C). This is consistent with the earlier observed higher sensitivity of the *paf1∆* cells to HU than to MMS^25,26^.

Genotoxins HU and MMS are known to cause replication stress/DNA damage via different mechanisms. As opposed to untreated controls, exposure to MMS (causes DNA damage via bases alkylation) resulted in a similar increase of the γ-H2A levels in both wild type and *paf1∆* cells (Fig. 4C,D), implying a Paf1-independent DNA damage by the MMS. Surprisingly, as compared to untreated controls, the γ-H2A levels upon HU exposure (inhibits replication via dNTPs depletion) show either a loss (mostly wild type cells) or no change (mostly *paf1∆* cells) in a gene-specific manner (Fig. 4C,D). These data show that Paf1 has a role in countering DNA damage caused by HU but not MMS toxicity on the pol III-transcribed tRNA genes. The results imply that Paf1 is not associated with replication-independent DNA damage.

### Paf1 counters the replication stress at the tRNA genes in a gene-specific manner

Loss of γ-H2A is reported to signify DNA repair at low levels of damage^54^. In the untreated wild type cells, low γ-H2A levels (Supplementary Fig. S4A) signify only a minimal and gene-specific DNA damage at the pol III-transcribed genes. Exposure of the wild type cells to HU inhibits replication and represses pol III transcription^55^. Hence reduced replication and transcription upon HU exposure may enable repair and reversal of the pre-existing low-level DNA damage (Supplementary Fig. S4A), resulting in observed loss of the γ-H2A levels (Fig. 4C).

With the slow replication and replication fork pausing, the DNA polymerase may be found at the genes in the normally cycling cells. An earlier, low-resolution microarray study had found enrichment of DNA pol2 at highly transcribed genomic sites, including three tRNA genes in the unsynchronized wild type cells^56^. Rrm3 is a DNA helicase component of replisome, which inhibits replication fork pausing at sites of stable DNA-protein complexes^56^. It was earlier found to facilitate fork passing past the transcription complex on the tRNA gene bodies^11^. The DNA pol2 increase on several tRNA genes in *rrm3* cells was found correlated with replication fork pausing^56^. Therefore, we estimated DNA pol 2 levels on the tRNA genes in the wild type and *paf1∆* cells as a measure of the fork stalling. As expected, our ChIP measurements found only low DNA pol2 levels in the normally cycling cells, which increased on most of the tested genes after exposure to 200 mM HU, in a gene-specific manner (Fig. 4E). In the *paf1∆* cells, the γ-H2A levels on most of the genes do not show loss upon HU-exposure (Fig. 4D), probably because the reported 2 fold decrease of *RNR1* expression^25,36^ causes additional replication stress on the genes in the untreated *paf1∆* cells (cf. Fig. 4C,D). Consistent with this, in the absence of any genotoxin, the pol2 levels on different genes in the *paf1∆* cells were found at levels either similar to or higher than the wild type levels (Supplementary Fig. S4D). Therefore, the pol2 increase with HU exposure on each gene in *paf1∆* cells was only half of the increase in the wild type cells (cf. Fig. 4E,F). Nevertheless, excepting *SCR1*, the pol2 levels in the *paf1∆* cells increase to almost the same level as seen in the wild type cells upon HU exposure (cf. Fig. 4E,F). Together, these results show that influences of Paf1 and HU on the replication stress on the tRNA genes are opposite and Paf1 plays a damage-preventive role on the tRNA genes during the replication fork progression. The observed increase of γ-H2A levels even in *paf1∆* cells, due to the chemically induced DNA damage by MMS (Fig. 4D) matches with this conclusion.

### Loss of Pol III occupancy upon replication stress requires Paf1

**Figure 5:**
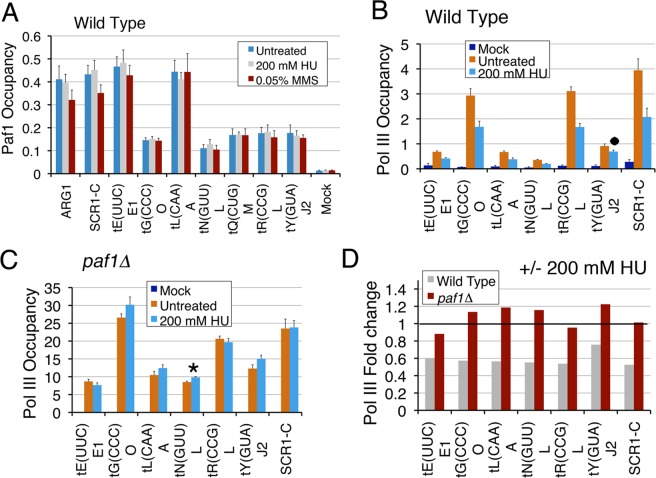
Pol III is lost from the genes under replication stress in a gene-specific manner. (**A**) Paf1 occupancy on the tested genes in the wild type cells does not change when exposed to the genotoxins HU or MMS. (**B**) Pol III occupancies in normally cycling wild type cells decrease upon exposure to HU. The dot marks the gene with insignificant decrease (p value = 0.29). (**C**) In the *paf1Δ* cells, pol III occupancies are not affected upon exposure to HU, except on *tN(GUU)L* (p value = 0.045), marked with an asterisk. (**D**) Data in the panel B for wild type and panel C for the *paf1Δ* cells were used to obtain the ratios HU treated/untreated cells. As a value of 1.0 (marked with a horizontal line) denotes no change, ratios on the y axis reveal that HU causes ~40% loss of pol III in the wild type but no change in the *paf1Δ* cells.

Unlike pol II-transcribed genes^26^, the Paf1 occupancy on the tested pol III-transcribed genes does not change (Fig. 5A) when wild type cells are exposed to the genotoxins. Exposure of the wild type cells to HU results in quick eviction of pol II from the replication-transcription encounter sites at pol II-transcribed genes^26^. We measured pol III levels on the genes when exposed to genotoxins. Consistent with earlier reported repression of the tRNA gene transcription under replication stress^55^, the pol III levels dropped significantly when wild type cells were exposed to genotoxins HU or MMS (Figs 5B and S5A, respectively). Similar to wild type cells, the pol III levels in the genotoxin-sensitive *paf1∆* cells drop when exposed to 0.05% MMS (Supplementary Fig. S5B).

In contrast to MMS effect, the pol III levels in the *paf1∆* cells do not decrease or alter on exposure to HU (Fig. 5C). We compared the fold decrease in pol III levels upon genotoxin exposure by calculating the ratios of the corresponding pol III levels on each gene in the *paf1∆* and wild type cells (Figs 5D and S5C). Consistent with the results so far, similar pol III decrease for both types of cells is found when exposed to MMS (Supplementary Fig. S5C), confirming MMS toxicity is not Paf1-dependent. In comparison, as compared to the *paf1∆* cells, HU exposure reduces pol III levels more in the wild type cells (Fig. 5D). We conclude that Paf1 is required to reduce the pol III levels in order to counter the replication stress on the pol III-transcribed genes, which could be one of the reasons behind the increased sensitivity of *paf1∆* cells to HU^25,26^.

### Pol III occupancy is highly increased upon Paf1 deletion

**Figure 6:**
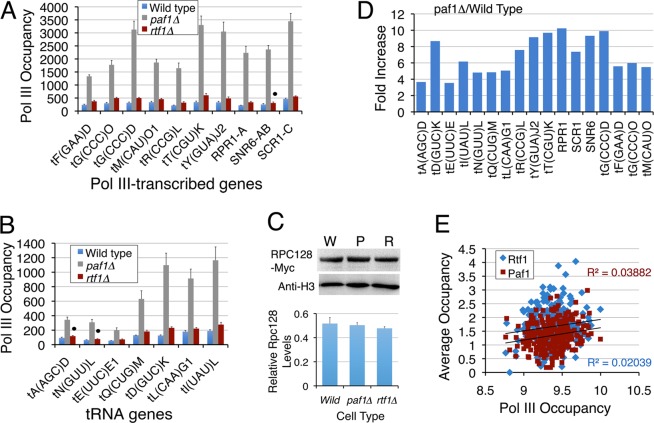
Pol III occupancy shows gene-specific increases in the *paf1Δ* and *rtf1Δ* cells. (**A**,**B**) Rpc128 occupancy on the pol III-transcribed genes relative to TelVIR region in wild type, *rtf1Δ* and *paf1Δ* cells. Averages and scatters from three biological replicates are plotted. The dot denotes that the change is non-significant in *rtf1∆* cells, probably due to higher scatter. (**C**) Western quantification of the total levels of Rpc128 protein in the wild type (W), *PAF1* (P) or *RTF1* (R) deletion cells. Lower panel shows that the Rpc128 protein levels normalized against H3 do not differ in the three types of cells. (**D**) Fold increase of pol III occupancy in the *paf1Δ cells* on the individual genes was calculated by using the data in the panels A and B. (**E**) Paf1, Rtf1^34^ and pol III^40^ occupancies on the tRNA genes are compared. Occupancies of Paf1 and Rtf1 (at −205 to +155 bp) do not show any correlation with pol III levels (at −200 to +300 bp).

We found the yeast pol III level was highly increased on all the tested genes in the *paf1∆* and all but three of the tested genes in the *rtf1∆* yeast cells (Fig. 6A,B). The total pol III levels (Rpc128 subunit) remain at the wild type levels in the *paf1∆* and *rtf1∆* cells (Fig. 6C). The pol III occupancy is highly affected by Paf1 deletion (~3.5–10.2 fold increase, Fig. 6D). In comparison, Rtf1 deletion causes only ~1.2–1.8 fold increase in pol III occupancy (Fig. 6A,B), which agrees with no change in the RNA levels in the *rtf1∆* cells (Supplementary Fig. S3D). Rtf1 was similarly found to show only a minor repressive effect on pol II-transcribed *SER3* expression earlier^38^. Paf1 and Rtf1 show unequal distributions (Figs 1, S1 and S2C) on the individual tRNA genes in the wild type cells. A comparison of average Paf1 and Rtf1 levels on the genes showed only a poor correlation with the pol III levels (Fig. 6E), although the correlation is better for Paf1 (R = 0.1455) than for Rtf1 (R = 0.0618). Consistent with the above results, the pol III occupancy on different genes is elevated to different levels in the *paf1∆* cells (Fig. 6A,B), indicating a gene-specific variation in Paf1 influence on the pol III levels. However, the high increase of pol III levels in *paf1∆* cells does not translate into a similar increase in tRNA levels.

### Gene-specific Paf1 effects are not due to spatial locations of the tRNA genes

**Figure 7:**
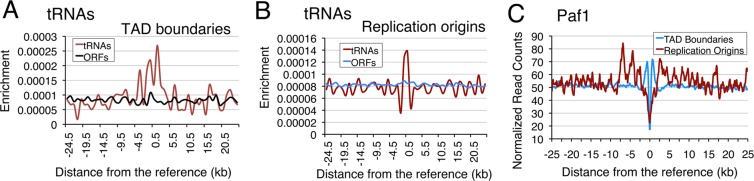
Paf1 is enriched around TAD boundaries. The normalized average enrichment across all the origins and TAD boundaries are shown. The bin sizes of 1000 bp and 100 bp for calculating the enrichment of tRNA start sites and Paf1 ChIP-exo reads respectively were used. Zero as reference point represents either the TAD boundary or the replication origin. (**A**,**B**) ORF enrichment was taken as a control. Average enrichment across (**A**) TAD boundaries and (**B**) replication origins was further normalized by the total TSS counts of tRNAs (299) and ORFs (6621), to bring the curves at comparable scale. (**C**) The average Paf1 enrichment (Normalized Read counts) across all the origins and TAD boundaries are shown.

Above results show that the major role of Paf1 on pol III-transcribed genes is to counter the replication-stress. Depending on the firing rate of an active replication origin, pausing of the replication fork on a genomic site may be higher in a proximal than the distal region. Similarly, the gene-specific occupancies and effects of Paf1 could be related to possible inclusion of the target genes in different, independently-acting chromosome domains. Therefore, we looked for proximity of tRNA genes and Paf1-binding sites to replication origins and TAD (Topologically associated domain) boundaries, which were reported to mark the transition between early and late replicating genomic regions in mouse and human cells^57^. Our analyses of the existing data in the public domain^58,59^ found 234 genes within 1 kb of TAD boundaries (Table S1A) and 47 genes within 2 kb of the replication origins (Table S1B). A summary of these results shows gene-specific differences in proximity of the tRNA genes to replication origins and TAD boundaries (Table S1). The results suggest that the tRNA genes exhibit significant enrichment around the replication origins (p-value = 0.0004) and the TAD boundaries (p-value = 0.02) when compared to the ORFs in the yeast genome (Figs 7A,B and S6A). Paf1 exhibited highly significant enrichment around the TAD boundaries (p = 4.8E-46) but not replication origins (Figs 7C and S6B). It may be worth noting here that one of the γ-H2A sites *tS(GCU)L*^12^ on which DNA pol2 levels increase in *rrm3* cells^56^, does not show proximity to either replication origin or a TAD boundary (Table S1). This indicates that replication fork stalling and DNA damage are not spatial location-dependent properties of the tRNA genes and gene-specific behaviours may be a culmination of various parameters like transcription activity, spatial location, association with different gene clusters and topological features of the genome etc. as evidenced by our earlier studies^60^.

In summary, this study has found that one of the known pol II-associated factors, the PAF1 complex occupies the tRNA genes in gene-specific manner. Most of its known roles on pol II-transcribed genes in transcription, nucleosome dynamics or histone methylations are not found on the pol III-transcribed genes. As the occupancies of Paf1/Rtf1 and pol III are not correlated, only a gene-specific change in the tRNA levels is found upon Paf1/Rtf1 deletion. The low levels of DNA pol2 and γ-H2A on the individual genes in the unsynchronized wild type cells increase upon genotoxins exposure and in the *paf1∆* cells, affirming retardation of replication on the genes under both the conditions. Pol III levels on the genes reduce upon genotoxins exposure while the pol III occupancy on the genes in the Paf1/Rtf1 deletion mutants shows high increase. This accumulation of pol III on the genes causes steric obstruction of the transcription and replication in the *paf1∆* cells. Taking together, this study shows a protective role of Paf1 on pol III-transcribed genes, which are naturally occurring sites of the replication fork stalling in the normally cycling cells.

## Discussion

High increase of pol III levels on the tRNA genes upon Paf1 deletion and its role in countering the replication stress on the pol III-transcribed genes are important findings in this study.

### Paf1 complex influences pol III transcription in a gene-specific manner

Paf1 complex has been reported to associate with and influence pol I and pol II transcription^18,19,28,36^. Gene-specific effects on transcription and pol III occupancy on different genes, tested in this study are similar to the earlier studies reporting a decrease in the transcript levels with increase/no change in gene-occupancy levels of pol I^18^ and differential effects on pol II transcript levels in the *paf1∆* cells^28^. The pol III-transcribed genes generally do not follow a single, common pattern of response under a condition and most of the times respond in a gene-specific manner^61^. In this context, we found one tRNA gene *tE(UUC)E1* shows highest Paf1 and lowest pol III occupancy among all the tested tRNA genes. But changes in the pol III levels in the *paf1∆* and *rtf1∆* cells and Paf1 level under repression are least on the *tE(UUC)E1* gene. Paf1 levels on the pol II-transcribed genes are reported to vary independent of its role in elongation^62^. Paf1 levels higher than a low, basal optimum required for transcription elongation serve regulatory roles whereby the gene-specific variations decide the fate of the transcripts^62^. Similar to this, we find different Paf1 levels influence the different pol III-transcribed genes in a gene-specific manner. Paf1 loss under starvation from most of the high but not low occupancy pol III-transcribed genes implies higher gene-specific Paf1 association under normal growth conditions. It may be noticeable that loss of Paf1 under starvation is not associated with increase of pol III levels as pol III is lost from all the genes under starvation^40,63^. Highly increased pol III levels on the tRNA genes upon Paf1 deletion may be the reason that no loss in pol III levels are seen upon HU exposure in the *paf1∆* cells. The presence of pol III TC components on the tRNA gene bodies protects them against the MNase (micrococcal nuclease) digestions^61,64^. Therefore, the increase in pol III levels at the gene body may be the reason for the apparent increase in intensity on the gene body in the MNase digestion profile of the chromatin in the *paf1∆* cells.

### PAF1C protects tRNA genes against replications stress-associated DNA damage

Paf1 deletion is reported to impair replication fork progression on the pol II-transcribed genes^26^. The increased DNA pol2 levels upon HU exposure and in the *paf1∆* cells denote replication stress and slowing down of replication fork at tRNA genes; conditions under which an increase in γ-H2A levels is seen with replication fork stalling^12^. The opposite effects of Paf1 and HU on the replication stress on the tRNA genes imply a protective role of Paf1 on the tRNA genes. The tRNA genes are non-randomly co-localized near TAD boundaries and replication origins. Consistent with the above, highly significant association of Paf1 with TAD boundaries suggests Paf1 may protect the tRNA genes near replication origins from replication stress and facilitate transition between early and late replication phases for the tRNA genes enriched around TAD boundaries under different conditions. Our results show that reducing the pol III occupancy is a cellular response to the replication stress caused by the genotoxins in order to reduce the transcription rate^55^ and facilitate the replication fork progression on the tRNA genes. Pol III TC and transcribed genes are potentially highly active and transcription to their full capacity may be an extravagance for cellular energy. The high transcription activity also makes them vulnerable to replication stress-induced DNA damage^10^. High accumulation of pol III on these short genes may also increase the steric clashes between the movements of the DNA and RNA polymerases, resulting in the increased transcription-replication conflict on the genes^8^. Paf1 intervention by restricting pol III levels on the genes resolves this as well.

PAF1C cooperates with Mec1 and INO80 in dislodging pol II from the sites of stalled replication fork under replication stress^26^. Deletion of the adaptor protein in the replication stress pathway, Mrc1 was found to show an effect similar to Paf1 on pol III occupancy and primary transcript synthesis on some of the tRNA genes^55^. On the tested pol III-transcribed genes in this study, increased levels of pol III, DNA pol2 and γ-H2A in the absence of PAF1C suggest that in the wild type cells PAF1C normally displaces pol III at the tRNA genes, which are known sites of high transcription activity, replication fork stalling and genomic fragility^10,11,65^.

### Absence of the Paf1 complex leads to the pol III stalling

A lack of correlation in occupancies of pol III with Paf1 and Rtf1 suggests PAF1C is not recruited by pol III. Its interaction with TFIIIC and TFIIIB and peak occupancy at the −10 bp position suggest it is recruited to the genes by TFIIIB or TFIIIC, probably before or simultaneous with the joining of pol III into the TC. At this stage, the low and gene-specific Paf1 occupancy suggests a gene-specific mechanism for a regulated recruitment or occupancy of Paf1 on the pol III-transcribed genes. On the other hand, the increased pol III levels on the tRNA genes may represent its increased recruitment or stalling in the *paf1∆* cells. Higher pol III recruitment on the genes would increase transcription while stalling leads to reduced transcription. Thus, our results showing only a small change in the tRNA levels with high increase of pol III are consistent with pol III stalling rather than increased recruitment in the *paf1∆* and *rtf1∆* cells. In this context, the earlier reported average occupancy profiles of pol III on all the tRNA genes; the highest pol III levels at the + 40 bp position^40^ and two short shoulder peaks at the beginning of the A and B boxes^53^, are consistent with the transient pol III pausing near the A box in the wild type cells. This implies that the increased pol III occupancy is seen due to the increased pol III pausing and therefore stalling in the *paf1∆* cells.

### A possible mechanism of pol III pause release by Paf1

The human Paf1 is reported to regulate the release of paused pol II levels at the 5′ gene-ends^21,66,67^, by both promoting and reducing the pol II pausing^21^ on the genes. The recruitment of Paf1 facilitates the Ser2 phosphorylation of pol II, resulting in release of paused pol II into elongation mode. When Paf1 is depleted, the paused pol II levels at the 5′-gene-ends increase^67^. Pol III is not known to undergo phosphorylation-dependent initiation-elongation mode switching, no factors equivalent to those participating in this switching of pol II are known to affect pol III transcription and pol III-transcribed genes are too short to differentiate between the two modes. Therefore, the PAF1C association with the pol III TC and localization near the TSS in the wild type cells could have a steric influence on the pol III occupancy. Thus, it could facilitate the pol III pause as well as its release near the A box, seen earlier in the genome-wide studies^40,53^. While TFIIIC bound to the intragenic boxes A and B may cause the transient pausing of pol III at all the genes^53,68^; the Paf1 presence may decrease the pausing near the TSS of the genes in a gene-specific manner.

The requirement of yeast Rtf1 for recruitment of PAF1C on the pol II-transcribed genes^69^ suggests it may be located peripherally in the complex serving possibly as the anchor subunit of the PAF1C^15,69^. Thus, employing Rtf1, Paf1 may hold pol III near the TSS during its transient pausing, observed earlier near the A box in the wild type cells^53^. Accordingly, only Rtf1 subunit was found associated with the pol III in transcriptionally active state^27^. Only a mild Rtf1 deletion effect on pol III levels is found probably because pol III is transiently held by other subunits at pause sites in the *rtf1∆* cells whereas this hold is released with loss of the PAF1C in the *paf1∆* cells^30^. This argument implies that in the absence of Paf1, normally high transcription rate and recycling of pol III^70,71^ result in the overloading and obstruction of the short gene body with pol III, eventually providing only a small gain in the transcription. Thus, similar to its earlier reported dual behaviour on the human pol II-transcribed genes^21^, Paf1 restricts the pol III levels on the tRNA genes by promoting both pause and release of pol III from the pause sites on these short pol III-transcribed genes. Our results are consistent with this dual gene-specific effect of Paf1 on pol III- transcribed genes.

By facilitating the pol III pausing, Paf1 regulates pol III advancement on the genes, which not only reduces pol III enrichment/occupancy and transcription but also allows timely clearance of the short gene body without affecting the high transcription frequency. On the other hand, releasing the paused pol III avoids the crowding of the short gene length with the transcribing pol III molecules, which may otherwise cause further obstruction to the replication fork progression on these naturally occurring replication fork pause sites. As opposed to this, when pol III is lost from the genes under repression^40,63^, Paf1 is also partially lost. Therefore, the significance of Paf1 association with the pol III TC is to keep pol III levels low on the tRNA genes, which are under replication stress even under the normal conditions^55^. Lowering the pol III levels may reduce the fork stalling, facilitate the fork progression and increase the genome integrity at individual gene loci. Thus, the inhibitory influence of PAF1C on the pol III pausing and replication stress-induced DNA damage on tRNA genes found in this study lay emphasis on a protective role for it in the wild type cells.

## Materials and Methods

### Yeast strains, plasmids, media and growth conditions

Yeast strains and primers used in this study are listed in the Tables S2 and S3 respectively. Cells were grown normally in YEP (yeast extract and peptone) medium containing 2% glucose at 30 °C. Cells were nutritionally deprived by shifting to 0.15X YEP without any carbon source after attaining the OD_600_ of 0.8 and allowed to grow for 2 more hrs at 30 °C, before harvesting. The genotoxin effect was followed by exposing the mid log phase cells to final concentration of 0.05% (v/v) MMS or 200 mM HU for 2 hours, before harvesting. Brf1 was FLAG-tagged according to Gelbart *et al*.^72^ and other taggings were performed by chromosomal integration of PCR amplified cassettes as described in Janke *et al*.^73^.

### Antibodies

Anti-FLAG antibody (F7425) and anti-FLAG M2 agarose beads (A2220) were purchased from Sigma. Anti-Myc tag and anti-HA tag antibodies used for ChIP and IP were from Millipore (05–724 and 05-904 respectively). Anti-myc, anti-HA and anti-FLAG antibodies used in western probings were from Santa Cruz ((9E10) SC-40 and (Y-11) SC-805) and Millipore (MAB3118) respectively. IgG sepharose beads were from GE Healthcare. Anti-H3 antibody (abcam 1791) and antibody against phosphorylated H2A S129 (ab15083) were from Abcam.

### Total Rpc128 protein level estimation

Cells carrying Myc tagged-*RPC128* gene were grown to logarithmic phase in YPD at 30 °C and whole cell lysate was prepared as previously described^74^. The lysate was resolved on SDS-PAGE, proteins were transferred to PVDF membrane and analysed by Western probing with anti-Myc and anti-H3 antibodies. Intensity of bands were measured using ImageJ software of Fuji. Average and scatter from three independent biological replicates was plotted.

### Chromatin Immunoprecipitation (ChIP) and Real Time PCR

ChIP and Real Time PCR estimations were performed as described earlier^75^. Cells were grown in YPD at 30 °C and fixed with 1% formaldehyde. Cells were washed thoroughly and lysed mechanically by glass beads in lysis buffer (50 mM K-HEPES pH 7.6, 1% Triton-X 100, 0.1% sodium deoxycholate, 2 mM EDTA, 150 mM NaCl) at 4 °C. Chromatin was sheared to average size of 300 bp by sonication and used for immunoprecipitation with corresponding antibodies (anti-Myc, anti-HA from Millipore) along with Protein A or Protein G agarose beads (GE Healthcare). Beads were washed in wash buffers and eluted at 65 °C in elution buffer (10 mM Tris-Cl pH 8, 2 mM EDTA, 200 mM NaCl, 1% SDS) for 30 min. The ChIP DNA was precipitated using ethanol after deproteinization with phenol, dissolved in TE and quantified in 10 µl reaction mixtures by a real-time PCR method based on SYBR green chemistry using Roche LC480 platform. The subtelomeric region of the right arm of the chromosome VI (TEL VIR) was used as a control region. Occupancies were calculated as the relative enrichment above the control values and normalized against the value for mock immunoprecipitation.

The “Fold enrichment method” was used to calculate the relative enrichments of DNA pol2 over the background using TelVIR as internal control region. In this method, the Ct values obtained from the ChIP and mock samples were first normalized with Ct values obtained from TelVIR region and then by the Ct values of Input^75^. Average and scatter from minimum three independent estimations are plotted. Genes showing non-significant changes with p value > 0.05 are marked with a dot wherever applicable. Rest of the genes have significant p values (<0.05).

### RNA isolation and quantification

Total RNA was isolated using acidic hot phenol method as previously described^74^ and Northern blotting was performed according to Karkusiewicz *et al*.^76^. Per lane 10 µg of total RNA was resolved on 15% Urea-PAGE and transferred from gel to Hybond N + membrane (GE Healthcare) by electroblotting in 1x TBE, cross-linked by UV radiation (1200 mJ/cm2). The membrane was pre-hybridized in prehyb/hyb buffer (2x SSC, 1X Denhardt’s solution, 0.2%SDS) and hybridized at 37 °C in the same solution with oligonucleotide probes (listed in the Table S2) labeled with [γ^32^P]-ATP by T4 polynucleotide kinase (New England Biolabs). The membrane was washed 3 times for 15 min with wash buffer (2X SSC and 0.2% SDS) at 42 °C and exposed to phosphorimaging screen (Fujifilm). Bands were visualized in phosphoimager (BIORAD) and intensities were quantified using Image Gauge software from Fuji. RNA levels were also estimated by the tRNA-HySeq method, essentially as described previously^52^, with the modifications as described under supplementary methods (Supplementary File S1).

### Genome-wide data analysis

The processed tab files containing forward and reverse tag counts, with their respective chromosomal location were downloaded from GSE83348 and used for the analysis of the ChIP-exo data^33^ on the occupancy of the PAF1C subunits on the tRNA genes as detailed under the Supplementary Information File S1.

All Raw data files for nucleosomal DNA^41^ from the wild type and *paf1∆* cells as treatment group and whole genome fragmented DNA as a control group were downloaded from NCBI GEO under accession no. GSE44879. The two sample analysis was performed on the nucleosomal occupancy data by employing Affymetrix Tiling Analysis Software (TAS) v1.1. Data were quantile normalized in pairs using only perfect match probes with a bandwidth of 30. Resulting bar files were used for Interval analysis with p-value threshold of 0.05, minimum run of 50 and maximum gap of 20 probes. Processed MNase-seq data files on histone methylations were downloaded under accession no GSE61888 from NCBI GEO. The plotHeatmap” function from deepTools was further used for mapping of respective histone methylations^49^ to yeast tRNA genes.

For calculating the tRNA and Paf1 enrichment around replication origins and TAD boundaries, the chromosomal coordinates of start sites for tRNAs and ORFs were obtained from SGD database (https://www.yeastgenome.org/). NGS reads for Paf1 ChIP-exo were obtained from GEO entry GSE83348^33^ and the counts for forward and reversed tags were summed. The replication origins and the TAD boundaries were taken from McCune *et al*.^58^ and Hsieh *et al*.^59^ respectively. We considered the ORF start sites as control and tested the enrichment of tRNAs and Paf1 by aggregating the counts in genomic bins around replication origins and TAD boundaries.

### Statistical analyses

Each measurement was repeated several times and average with scatter for minimum three biological replicates was taken for plotting the graphs. Statistical significance of a difference between the two values was calculated using two-tailed unpaired Student’s t-test. The insignificant changes (p > 0.05) if any, in every set of data are marked in every Figure panel. Unless otherwise stated, remaining all the comparisons are significant (p < 0.05) and not marked.

The p-values for the significant enrichment of tRNAs and Paf1 around replication origins and TAD boundaries were calculated by using two-tailed t-test of feature-proximal and the feature-distant bins. Feature proximity was guided by the trend in the enrichment plots of tRNA/Paf1 around origins and TAD boundaries. For the TAD boundaries, bins at +/−0.5 kb were considered proximal, while the bins at +/−2.5 kb were considered as distant considering the narrow range of enrichment. In case of replication origins, the bins spread within +/−1.5 kb were considered proximal while the ones at +/−5 kb were considered as distant due to broader enrichment range. The average values in the feature-proximal and the feature-distant bins were calculated and the resulting vectors were subjected to two-tailed t-test.

## Supplementary information

File S1

## Data Availability

The tRNA-HySeq datasets generated in this study have been deposited to NCBI GEO database (http://www.ncbi.nlm.nih.gov/geo/) under accession no. GSE134987.
