# Supplementary material for: Yeast PAF1 complex counters the pol III accumulation and replication stress on the tRNA genes
Source: Sci Rep. 2019 Sep 9;9:12892. doi: 10.1038/s41598-019-49316-5 (PMC6733944; doi:10.1038/s41598-019-49316-5)
Supplement: Supplementary file 1 — File S1 [file 41598_2019_49316_MOESM1_ESM.pdf]

Supplemental Material

for the manuscript

Yeast PAF1 complex counters the pol III accumulation and replication stress on the tRNA genes

Pratibha Bhalla[1,^], Ashutosh Shukla[1,^], Dipti Vinayak Vernekar[1,#], Aneeshkumar Gopalakrishnan Arimbasseri[2], Kuljeet Singh Sandhu[3], Purnima Bhargava[1,*]

[1] Centre for Cellular and Molecular Biology (Council of Scientific and Industrial Research), Hyderabad, India

[2] Molecular Genetics Laboratory, National Institute of Immunology New Delhi, India

[3] Department of Biological Sciences, Indian Institute of Science Education and Research (IISER) – Mohali, Manauli, India

List of the Contents of this File

I. Supplementary Methods

II. Supplementary Tables S1- S4

III. Supplementary References

IV. Supplementary Figure legends

V. Supplementary Figures S1-S6

I. Supplementary Methods

tRNA estimation using tRNA-HydroSeq (HySeq) method

The tRNAs were size selected from total RNA, followed by partial hydrolysis in 10 mM bicarbonate buffer (pH 9.8) at 90ºC for 5 minutes. The 19-35 nt RNA fragments were dephosphorylated after size-selecting on a 15% TBE-Urea-PAGE gel, followed by adapter ligation (universal miRNA cloning linker, NEB, S1315S). Adapter ligated fragments were size selected using a 12% TBE-Urea-PAGE gel and reverse transcribed using olMGL24. cDNA was purified on a 10% TBE-Urea-PAGE gel, followed by circularization and PCR amplification using a common forward primer and barcoded reverse primers (Table S4).

All data analysis was done using Galaxy platform (1). FastQC analysis was done with sequencing data followed by clipping of adapter sequences and trimming to remove the low quality bases. Resulting sequences were aligned to a genome file containing all unique tRNA sequences in *S. cerevisease*. After SAM to BAM conversion of the alignment file, the read counts were calculated using IdxStats Read counts were analyzed using Deseq package in bioconductor (2).

Genome-wide Data Analyses

The open source "Bio toolbox" from Timothy J. Parnell was used to map binding sites of all the subunits of Paf1 complex around the TSS of the tRNAs genes. The genome-wide occupancy of Rtf1 on the tRNA genes in the yeast genome was analysed also from the published ChIP-seq data (34). The reads uniquely aligned to sacCer3 yeast assembly with mapping quality scores >30 were processed for further analyses. The "bamCompare" function from deepTools package was used for Library size adjustments (SES method) and log_2 ratio of IP vs Input was calculated (3). The "plotProfile" and "plotHeatmap" functions in deepTools, "pheatmap" functions in R-package, and the in-house PERL and JAVA programs were used for plotting the average occupancy profiles and heatmaps (3).

We tested the enrichment of tRNAs and Paf1 by aggregating the counts in genomic bins around replication origins and TAD boundaries. We used the bin size of 1000 bp and 100 bp for the enrichment of tRNA start sites and Paf1 ChIP-exo reads respectively. We considered the ORF start sites as control. To bring the tRNA and ORF enrichment values at comparable scale, the average enrichment values were further normalized by total number of tRNA and ORF start sites respectively. Heatmaps were drawn using *pheatmap* R-package (https://cran.r-project.org/package=pheatmap).

II. Supplementary Tables

Table S1A: Number of tRNA genes within 1 kb of the TAD boundary/ replication origin

Type of tRNA	Genome Total	Proximal to TAD boundary	Proximal to replication origin	tRNA ID/tRNA position/Nearest TAD boundary	tRNA ID/tRNA position/Nearest origin
tP(UGG)	11	7	2	tP(UGG)A/1:139254/139182, tP(UGG)L/12:92650/92531, tP(UGG)N1/14:547196/547075, tP(UGG)N2/14:568217/567660, tP(UGG)O1/15:301198/301083, tP(UGG)O2/15:464551/464446, tP(UGG)F/6:101478/101364,	tP(UGG)L/12:92650/92000, tP(UGG)O2/15:464551/464000
tA(UGC)	6	4	0	tA(UGC)A/1:166339/165857, tA(UGC)O/15:854187/854260, tA(UGC)E/5:312023/311896, tA(UGC)G/7:794489/794473,	
tL(CAA)	10	9	1	tL(CAA)A/1:181254/181241, tL(CAA)K/11:458670/458550, tL(CAA)L/12:628497/628476, tL(CAA)M/13:505008/505032, tL(CAA)C/3:90972/90309, tL(CAA)D/4:1461715/1461719, tL(CAA)G1/7:205634/205579, tL(CAA)G2/7:423205/423230, tL(CAA)G3/7:857378/857420,	tL(CAA)D/4:1461715/1462000

tS(AGA)	11	8	1	tS(AGA)A/1:182522/182550,<br>tS(AGA)L/12:167944/167986,<br>tS(AGA)M/13:259158/259123,<br>tS(AGA)B/2:227156/227482,<br>tS(AGA)D1/4:437853/437913,<br>tS(AGA)D3/4:1305712/1305621,<br>tS(AGA)E/5:86685/86743,<br>tS(AGA)H/8:133026/133027,	tS(AGA)H/8:133026/133000,
tT(AGU)	11		2	tT(AGU)J/10:59100/58980,<br>tT(AGU)N1/14:104877/104925,<br>tT(AGU)O1/15:113874/113802,<br>tT(AGU)O2/15:354041/354428,<br>tT(AGU)C/3:295556/295562,<br>tT(AGU)D/4:434264/434269,<br>tT(AGU)H/8:116107/115593,<br>tT(AGU)I1/9:175103/175019,<br>tT(AGU)I2/9:325820/325746,	tT(AGU)N2/14:560693/561000<br>,<br>tT(AGU)O1/15:113874/114000
tE(UUC)	15	13	1	tE(UUC)J/10:115939/116070,<br>tE(UUC)K/11:141018/141035,<br>tE(UUC)L/12:797249/796856,<br>tE(UUC)M/13:290872/290820,<br>tE(UUC)P/16:210192/210176,<br>tE(UUC)B/2:645238/645171,<br>tE(UUC)C/3:82462/83046,<br>tE(UUC)E1/5:177170/177198,<br>tE(UUC)E2/5:355005/354961,<br>tE(UUC)E3/5:487331/487321,<br>tE(UUC)G1/7:328654/328571,<br>tE(UUC)G2/7:401527/402060,<br>tE(UUC)I/9:370488/370472,	tE(UUC)P/16:210192/211000,
tA(AGC)	11	7	0	tA(AGC)K1/11:219967/219902,<br>tA(AGC)M1/13:321147/321345,<br>tA(AGC)M2/13:768369/768436,<br>tA(AGC)P/16:856974/857284,<br>tA(AGC)D/4:410451/411089,<br>tA(AGC)F/6:204924/204910,<br>tA(AGC)G/7:774421/774364,	
tD(GUC)	17	14	3	tD(GUC)J1/10:204806/204825,<br>tD(GUC)J2/10:355527/354781,<br>tD(GUC)J3/10:374424/374577,<br>tD(GUC)J4/10:541579/541023,<br>tD(GUC)K/11:513332/513288,<br>tD(GUC)L1/12:427203/427177,<br>tD(GUC)L2/12:793989/794453,<br>tD(GUC)M/13:463625/463557,<br>tD(GUC)O/15:572029/572061,<br>tD(GUC)B/2:406031/405833,<br>tD(GUC)D/4:569035/568899,<br>tD(GUC)G1/7:531681/531712,<br>tD(GUC)G2/7:544577/544916,<br>tD(GUC)I2/9:336349/336331,	tD(GUC)J1/10:204806/204000,<br>tD(GUC)J3/10:374424/375000,<br>tD(GUC)L2/12:793989/794000

tR(ACG)	7	5	0	tR(ACG)J/10:234011/233999,<br>tR(ACG)K/11:491040/490802,<br>tR(ACG)L/12:374427/374372,<br>tR(ACG)O/15:340299/340394,<br>tR(ACG)D/4:619969/620044,	
tY(GUA)	9	7	1	tY(GUA)J1/10:354332/354781,<br>tY(GUA)J2/10:542956/543080,<br>tY(GUA)M2/13:838016/837936,<br>tY(GUA)O/15:288280/288229,<br>tY(GUA)D/4:946400/946275,<br>tY(GUA)F1/6:167525/167402,<br>tY(GUA)F2/6:210619/210362,	tY(GUA)F1/6:167525/168000,
tR(UCU)	12	11	1	tR(UCU)J1/10:355445/354781,<br>tR(UCU)J2/10:374506/374577,<br>tR(UCU)K/11:162487/162594,<br>tR(UCU)M2/13:131825/131806,<br>tR(UCU)M1/13:747892/748079,<br>tR(UCU)B/2:405949/405833,<br>tR(UCU)D/4:568953/568899,<br>tR(UCU)E/5:138737/138670,<br>tR(UCU)G1/7:405541/405581,<br>tR(UCU)G3/7:736411/736399,<br>tR(UCU)G2/7:828723/828744,	tR(UCU)J2/10:374506/375000,
tV(AAC)	14	12	1	tV(AAC)J/10:378433/378548,<br>tV(AAC)K1/11:308144/308450,<br>tV(AAC)K2/11:379753/379651,<br>tV(AAC)L/12:687859/687921,<br>tV(AAC)M2/13:420588/420635,<br>tV(AAC)M3/13:586636/586708,<br>tV(AAC)O/15:663812/663856,<br>tV(AAC)E1/5:438773/438825,<br>tV(AAC)E2/5:469530/469464,<br>tV(AAC)G3/7:73829/74188,<br>tV(AAC)G1/7:412294/412327,<br>tV(AAC)G2/7:823555/823922,	tV(AAC)E1/5:438773/439000,
tM(CAU)	12	8	0	tM(CAU)J1/10:391043/391188,<br>tM(CAU)J2/10:423009/422948,<br>tM(CAU)J3/10:517813/517837,<br>tM(CAU)M/13:572883/572963,<br>tM(CAU)O1/15:710272/710468,<br>tM(CAU)P/16:338848/339677,<br>tM(CAU)D/4:1175901/1175526,<br>tM(CAU)E/5:100204/100374,	

tG(GCC)	16	15	3	tG(GCC)J1/10:396726/396818,<br>tG(GCC)J2/10:531898/531837,<br>tG(GCC)O1/15:226611/226685,<br>tG(GCC)O2/15:282234/282130,<br>tG(GCC)P1/16:572339/572332,<br>tG(GCC)P2/16:860449/860391,<br>tG(GCC)B/2:197629/197687,<br>tG(GCC)C/3:142701/142837,<br>tG(GCC)D1/4:83618/83561,<br>tG(GCC)D2/4:992902/993115,<br>tG(GCC)E/5:61890/61800,<br>tG(GCC)F1/6:162298/162309,<br>tG(GCC)F2/6:180974/180929,<br>tG(GCC)G1/7:845719/845652,<br>tG(GCC)G2/7:930953/930976,	tG(GCC)M/13:183898/184000,<br>tG(GCC)O1/15:226611/227000<br>, tG(GCC)B/2:197629/198000,
tK(CUU)	14	11	0	tK(CUU)J/10:415038/414998,<br>tK(CUU)K/11:203071/203068,<br>tK(CUU)M/13:480621/480603,<br>tK(CUU)P/16:582134/582212,<br>tK(CUU)C/3:151284/151749,<br>tK(CUU)D1/4:1201822/1201763,<br>tK(CUU)D2/4:1352466/1352496,<br>tK(CUU)G1/7:122341/122298,<br>tK(CUU)G2/7:185714/185766,<br>tK(CUU)G3/7:876466/876458,<br>tK(CUU)I/9:300228/300313,	
tW(CCA)	6	6	0	tW(CCA)J/10:415931/415927,<br>tW(CCA)K/11:303023/302937,<br>tW(CCA)M/13:379303/379542,<br>tW(CCA)P/16:56169/55696,<br>tW(CCA)G1/7:287350/287451,<br>tW(CCA)G2/7:878710/878800,	
tL(UAA)	8	7	0	tL(UAA)J/10:424432/424382,<br>tL(UAA)K/11:84291/84202,<br>tL(UAA)L/12:962972/963297,<br>tL(UAA)N/14:726134/726158,<br>tL(UAA)B1/2:9666/10602,<br>tL(UAA)B2/2:347686/347679,<br>tL(UAA)D/4:519826/520479,	
tR(CCU)	1	1	0	tR(CCU)J/10:538626/538579,	
tL(UAG)	3	2	0	tL(UAG)J/10:618019/618030,<br>tL(UAG)L2/12:732190/732049,	
tT(CGU)	1	1	0	tT(CGU)K/11:46735/46809,	
tN(GUU)	11	7	0	tN(GUU)K/11:74697/74667,<br>tN(GUU)L/12:975983/975962,<br>tN(GUU)N2/14:632672/631919,<br>tN(GUU)O2/15:487512/487435,<br>tN(GUU)C/3:127789/127765,<br>tN(GUU)F/6:137486/137450,<br>tN(GUU)G/7:731210/731052,	

tH(GUG)	8	7	0	tH(GUG)K/11:313401/313461,<br>tH(GUG)M/13:363135/363208,<br>tH(GUG)E1/5:207428/207363,<br>tH(GUG)E2/5:434541/434574,<br>tH(GUG)G1/7:110625/110647,<br>tH(GUG)G2/7:319852/319509,<br>tH(GUG)H/8:62826/62736,	
tK(UUU)	8	6	0	tK(UUU)K/11:579060/579016,<br>tK(UUU)L/12:875471/875381,<br>tK(UUU)O/15:438738/438653,<br>tK(UUU)P/16:769207/769179,<br>tK(UUU)D/4:359672/359492,<br>tK(UUU)G1/7:115583/115786,	
tQ(UUG)	10	8	0	tQ(UUG)L/12:448650/447739,<br>tQ(UUG)B/2:350898/350869,<br>tQ(UUG)C/3:168301/169132,<br>tQ(UUG)D1/4:521043/520479,<br>tQ(UUG)D2/4:645153/644493,<br>tQ(UUG)D3/4:802731/802700,<br>tQ(UUG)E2/5:131082/131121,<br>tQ(UUG)E1/5:250357/250113,	
tI(UAU)	2	1	0	tI(UAU)L/12:605300/605336,	
tI(AAU)	13	12	2	tI(AAU)L1/12:734875/734956,<br>tI(AAU)L2/12:1052071/1052114,<br>tI(AAU)N1/14:569867/569944,<br>tI(AAU)N2/14:602385/602380,<br>tI(AAU)P1/16:819602/819489,<br>tI(AAU)P2/16:880296/880363,<br>tI(AAU)B/2:197567/197687,<br>tI(AAU)D/4:668007/668066,<br>tI(AAU)E2/5:551285/551372,<br>tI(AAU)G/7:739195/738849,<br>tI(AAU)I1/9:183440/183526,<br>tI(AAU)I2/9:210738/210748,	tI(AAU)P1/16:819602/819000,<br>tI(AAU)B/2:197567/198000,
tX(XXX)	2	2	0	tX(XXX)L/12:784453/784388,<br>tX(XXX)D/4:1150842/1150695,	
tR(CCG)	1	1	0	tR(CCG)L/12:818680/819067,	
tF(GAA)	11	8	0	tF(GAA)M/13:352370/352338,<br>tF(GAA)N/14:374959/374930,<br>tF(GAA)P1/16:560198/560168,<br>tF(GAA)P2/16:622540/622615,<br>tF(GAA)B/2:36488/36564,<br>tF(GAA)F/6:157916/157912,<br>tF(GAA)H1/8:237848/237986,<br>tF(GAA)H2/8:358478/358510,	
tQ(CUG)	1	1	0	tQ(CUG)M/13:808246/808340,	
tG(UCC)	4	1	0	tG(UCC)G/7:779687/779631,	
tP(AGG)	2	2	0	tP(AGG)N/14:631846/631919,<br>tP(AGG)C/3:123577/123564,	

tS(GCU)	3	2	0	tS(GCU)O/15:274773/274755, tS(GCU)F/6:191513/191585,	
tG(CCC)	2	1	0	tG(CCC)D/4:1257008/1257068,	
tC(GCA)	5	4	0	tC(GCA)P1/16:435893/435877, tC(GCA)P2/16:775765/775808, tC(GCA)B/2:643007/643075, tC(GCA)G/7:707108/706939,	
tS(UGA)	4	3	1	tS(UGA)P/16:689646/689590, tS(UGA)E/5:288443/288461, tS(UGA)I/9:248931/249351,	tS(UGA)E/5:288443/288000,
tT(UGU)	5	4	0	tT(UGU)P/16:744355/744348, tT(UGU)G1/7:661820/661761, tT(UGU)G2/7:1004287/1004284, tT(UGU)H/8:467061/466970,	
tW(UCA)	1	0	0		
tI(GAU)	1	0	0		
tT(UAG)	1	0	0		
tV(UAC)	3	2	1	tV(UAC)B/2:326792/326875, tV(UAC)D/4:488797/488806,	tV(UAC)B/2:326792/326000,
tS(CGA)	1	1	0	tS(CGA)C/3:228042/228871,	
tE(CUC)	2	2	1	tE(CUC)D/4:1017207/1017272, tE(CUC)I/9:197663/197748,	tE(CUC)D/4:1017207/1017000 ,
tV(CAC)	2	2	0	tV(CAC)D/4:1075472/1075481, tV(CAC)H/8:475706/475813,	
tL(GAG)	1	0	0		
**Total**	**299**	**234**	**21**		

Table S1B: Number of tRNA genes within 2 kb of the TAD boundary/ replication origin

Type of tRNA	Genome Total	Proximal to TAD boundary	Proximal to replication origin	tRNA ID/tRNA position/Nearest TAD boundary	tRNA ID/tRNA position/Nearest origin
tP(UGG)	11	9	3	tP(UGG)A/1:139254/139182, tP(UGG)L/12:92650/92531, tP(UGG)N1/14:547196/547075, tP(UGG)N2/14:568217/567660, tP(UGG)O1/15:301198/301083, tP(UGG)O2/15:464551/464446, tP(UGG)O3/15:980787/979621, tP(UGG)F/6:101478/101364, tP(UGG)H/8:388893/389961,	tP(UGG)L/12:92650/92000, tP(UGG)O2/15:464551/464000 , tP(UGG)O3/15:980787/982000

tA(UGC)	6	4	0	tA(UGC)A/1:166339/165857, tA(UGC)O/15:854187/854260, tA(UGC)E/5:312023/311896, tA(UGC)G/7:794489/794473,	
tL(CAA)	10	10	2	tL(CAA)A/1:181254/181241, tL(CAA)K/11:458670/458550, tL(CAA)L/12:628497/628476, tL(CAA)M/13:505008/505032, tL(CAA)N/14:443119/444603, tL(CAA)C/3:90972/90309, tL(CAA)D/4:1461715/1461719, tL(CAA)G1/7:205634/205579, tL(CAA)G2/7:423205/423230, tL(CAA)G3/7:857378/857420,	tL(CAA)D/4:1461715/1462000 , tL(CAA)G1/7:205634/204000,
tS(AGA)	11	10	2	tS(AGA)A/1:182522/182550, tS(AGA)J/10:524012/525826, tS(AGA)L/12:167944/167986, tS(AGA)M/13:259158/259123, tS(AGA)B/2:227156/227482, tS(AGA)D1/4:437853/437913, tS(AGA)D2/4:980974/979192, tS(AGA)D3/4:1305712/1305621, tS(AGA)E/5:86685/86743, tS(AGA)H/8:133026/133027,	tS(AGA)D1/4:437853/436000, tS(AGA)H/8:133026/133000,
tT(AGU)	11	11	3	tT(AGU)J/10:59100/58980, tT(AGU)N1/14:104877/104925, tT(AGU)N2/14:560693/559297, tT(AGU)O1/15:113874/113802, tT(AGU)O2/15:354041/354428, tT(AGU)B/2:266450/267490, tT(AGU)C/3:295556/295562, tT(AGU)D/4:434264/434269, tT(AGU)H/8:116107/115593, tT(AGU)I1/9:175103/175019, tT(AGU)I2/9:325820/325746,	tT(AGU)N2/14:560693/561000 , tT(AGU)O1/15:113874/114000 , tT(AGU)D/4:434264/436000,
tE(UUC)	15	13	3	tE(UUC)J/10:115939/116070, tE(UUC)K/11:141018/141035, tE(UUC)L/12:797249/796856, tE(UUC)M/13:290872/290820, tE(UUC)P/16:210192/210176, tE(UUC)B/2:645238/645171, tE(UUC)C/3:82462/83046, tE(UUC)E1/5:177170/177198, tE(UUC)E2/5:355005/354961, tE(UUC)E3/5:487331/487321, tE(UUC)G1/7:328654/328571, tE(UUC)G2/7:401527/402060, tE(UUC)I/9:370488/370472,	tE(UUC)J/10:115939/114000, tE(UUC)P/16:210192/211000, tE(UUC)E2/5:355005/354000,

tA(AGC)	11	10	1	tA(AGC)J/10:197313/198587, tA(AGC)K1/11:219967/219902, tA(AGC)K2/11:518060/519188, tA(AGC)L/12:656934/658409, tA(AGC)M1/13:321147/321345, tA(AGC)M2/13:768369/768436, tA(AGC)P/16:856974/857284, tA(AGC)D/4:410451/411089, tA(AGC)F/6:204924/204910, tA(AGC)G/7:774421/774364,	tA(AGC)K2/11:518060/517000 ,
tD(GUC)	17	15	5	tD(GUC)J1/10:204806/204825, tD(GUC)J2/10:355527/354781, tD(GUC)J3/10:374424/374577, tD(GUC)J4/10:541579/541023, tD(GUC)K/11:513332/513288, tD(GUC)L1/12:427203/427177, tD(GUC)L2/12:793989/794453, tD(GUC)M/13:463625/463557, tD(GUC)O/15:572029/572061, tD(GUC)B/2:406031/405833, tD(GUC)D/4:569035/568899, tD(GUC)G1/7:531681/531712, tD(GUC)G2/7:544577/544916, tD(GUC)I1/9:324303/325746, tD(GUC)I2/9:336349/336331,	tD(GUC)J1/10:204806/204000, tD(GUC)J3/10:374424/375000, tD(GUC)J4/10:541579/540000, tD(GUC)L2/12:793989/794000 , tD(GUC)B/2:406031/408000,
tR(ACG)	7	6	1	tR(ACG)J/10:234011/233999, tR(ACG)K/11:491040/490802, tR(ACG)L/12:374427/374372, tR(ACG)O/15:340299/340394, tR(ACG)D/4:619969/620044, tR(ACG)E/5:492352/491028,	tR(ACG)L/12:374427/373000,
tY(GUA)	9	8	1	tY(GUA)J1/10:354332/354781, tY(GUA)J2/10:542956/543080, tY(GUA)M1/13:168883/169932, tY(GUA)M2/13:838016/837936, tY(GUA)O/15:288280/288229, tY(GUA)D/4:946400/946275, tY(GUA)F1/6:167525/167402, tY(GUA)F2/6:210619/210362,	tY(GUA)F1/6:167525/168000,
tR(UCU)	12	11	1	tR(UCU)J1/10:355445/354781, tR(UCU)J2/10:374506/374577, tR(UCU)K/11:162487/162594, tR(UCU)M2/13:131825/131806, tR(UCU)M1/13:747892/748079, tR(UCU)B/2:405949/405833, tR(UCU)D/4:568953/568899, tR(UCU)E/5:138737/138670, tR(UCU)G1/7:405541/405581, tR(UCU)G3/7:736411/736399, tR(UCU)G2/7:828723/828744,	tR(UCU)J2/10:374506/375000,

tV(AAC)	14	14	2	tV(AAC)J/10:378433/378548, tV(AAC)K1/11:308144/308450, tV(AAC)K2/11:379753/379651, tV(AAC)L/12:687859/687921, tV(AAC)M1/13:372445/370654, tV(AAC)M2/13:420588/420635, tV(AAC)M3/13:586636/586708, tV(AAC)O/15:663812/663856, tV(AAC)E1/5:438773/438825, tV(AAC)E2/5:469530/469464, tV(AAC)G3/7:73829/74188, tV(AAC)G1/7:412294/412327, tV(AAC)G2/7:823555/823922, tV(AAC)H/8:85298/86987,	tV(AAC)M1/13:372445/371000, tV(AAC)E1/5:438773/439000,
tM(CAU)	12	9	0	tM(CAU)J1/10:391043/391188, tM(CAU)J2/10:423009/422948, tM(CAU)J3/10:517813/517837, tM(CAU)M/13:572883/572963, tM(CAU)O1/15:710272/710468, tM(CAU)P/16:338848/339677, tM(CAU)C/3:149991/148413, tM(CAU)D/4:1175901/1175526, tM(CAU)E/5:100204/100374,	
tG(GCC)	16	15	3	tG(GCC)J1/10:396726/396818, tG(GCC)J2/10:531898/531837, tG(GCC)O1/15:226611/226685, tG(GCC)O2/15:282234/282130, tG(GCC)P1/16:572339/572332, tG(GCC)P2/16:860449/860391, tG(GCC)B/2:197629/197687, tG(GCC)C/3:142701/142837, tG(GCC)D1/4:83618/83561, tG(GCC)D2/4:992902/993115, tG(GCC)E/5:61890/61800, tG(GCC)F1/6:162298/162309, tG(GCC)F2/6:180974/180929, tG(GCC)G1/7:845719/845652, tG(GCC)G2/7:930953/930976,	tG(GCC)M/13:183898/184000, tG(GCC)O1/15:226611/227000, tG(GCC)B/2:197629/198000,
tK(CUU)	14	12	2	tK(CUU)J/10:415038/414998, tK(CUU)K/11:203071/203068, tK(CUU)M/13:480621/480603, tK(CUU)P/16:582134/582212, tK(CUU)C/3:151284/151749, tK(CUU)D1/4:1201822/1201763, tK(CUU)D2/4:1352466/1352496, tK(CUU)E2/5:435752/434574, tK(CUU)G1/7:122341/122298, tK(CUU)G2/7:185714/185766, tK(CUU)G3/7:876466/876458, tK(CUU)I/9:300228/300313,	tK(CUU)J/10:415038/417000, tK(CUU)D2/4:1352466/1354000,
tW(CCA)	6	6	3	tW(CCA)J/10:415931/415927, tW(CCA)K/11:303023/302937, tW(CCA)M/13:379303/379542, tW(CCA)P/16:56169/55696, tW(CCA)G1/7:287350/287451, tW(CCA)G2/7:878710/878800,	tW(CCA)J/10:415931/417000, tW(CCA)K/11:303023/302000, tW(CCA)G1/7:287350/286000,

tL(UAA)	8	7	0	tL(UAA)J/10:424432/424382,<br>tL(UAA)K/11:84291/84202,<br>tL(UAA)L/12:962972/963297,<br>tL(UAA)N/14:726134/726158,<br>tL(UAA)B1/2:9666/10602,<br>tL(UAA)B2/2:347686/347679,<br>tL(UAA)D/4:519826/520479,	
tR(CCU)	1	1	1	tR(CCU)J/10:538626/538579,	tR(CCU)J/10:538626/540000,
tL(UAG)	3	2	0	tL(UAG)J/10:618019/618030,<br>tL(UAG)L2/12:732190/732049,	
tT(CGU)	1	1	0	tT(CGU)K/11:46735/46809,	
tN(GUU)	11	8	1	tN(GUU)K/11:74697/74667,<br>tN(GUU)L/12:975983/975962,<br>tN(GUU)N2/14:632672/631919,<br>tN(GUU)O1/15:228404/226685,<br>tN(GUU)O2/15:487512/487435,<br>tN(GUU)C/3:127789/127765,<br>tN(GUU)F/6:137486/137450,<br>tN(GUU)G/7:731210/731052,	tN(GUU)O1/15:228404/22700<br>0,
tH(GUG)	8	7	1	tH(GUG)K/11:313401/313461,<br>tH(GUG)M/13:363135/363208,<br>tH(GUG)E1/5:207428/207363,<br>tH(GUG)E2/5:434541/434574,<br>tH(GUG)G1/7:110625/110647,<br>tH(GUG)G2/7:319852/319509,<br>tH(GUG)H/8:62826/62736,	tH(GUG)G1/7:110625/112000,
tK(UUU)	8	6	1	tK(UUU)K/11:579060/579016,<br>tK(UUU)L/12:875471/875381,<br>tK(UUU)O/15:438738/438653,<br>tK(UUU)P/16:769207/769179,<br>tK(UUU)D/4:359672/359492,<br>tK(UUU)G1/7:115583/115786,	tK(UUU)O/15:438738/437000,
tQ(UUG)	10	9	2	tQ(UUG)L/12:448650/447739,<br>tQ(UUG)B/2:350898/350869,<br>tQ(UUG)C/3:168301/169132,<br>tQ(UUG)D1/4:521043/520479,<br>tQ(UUG)D2/4:645153/644493,<br>tQ(UUG)D3/4:802731/802700,<br>tQ(UUG)E2/5:131082/131121,<br>tQ(UUG)E1/5:250357/250113,<br>tQ(UUG)H/8:134392/133027,	tQ(UUG)C/3:168301/167000,<br>tQ(UUG)H/8:134392/133000,
tI(UAU)	2	1	0	tI(UAU)L/12:605300/605336,	

tI(AAU)	13	13	2	tI(AAU)L1/12:734875/734956,<br>tI(AAU)L2/12:1052071/1052114,<br>tI(AAU)N1/14:569867/569944,<br>tI(AAU)N2/14:602385/602380,<br>tI(AAU)P1/16:819602/819489,<br>tI(AAU)P2/16:880296/880363,<br>tI(AAU)B/2:197567/197687,<br>tI(AAU)D/4:668007/668066,<br>tI(AAU)E1/5:443202/441847,<br>tI(AAU)E2/5:551285/551372,<br>tI(AAU)G/7:739195/738849,<br>tI(AAU)I1/9:183440/183526,<br>tI(AAU)I2/9:210738/210748,	tI(AAU)P1/16:819602/819000,<br>tI(AAU)B/2:197567/198000,
tX(XXX)	2	2	0	tX(XXX)L/12:784453/784388,<br>tX(XXX)D/4:1150842/1150695,	
tR(CCG)	1	1	0	tR(CCG)L/12:818680/819067,	
tF(GAA)	11	8	1	tF(GAA)M/13:352370/352338,<br>tF(GAA)N/14:374959/374930,<br>tF(GAA)P1/16:560198/560168,<br>tF(GAA)P2/16:622540/622615,<br>tF(GAA)B/2:36488/36564,<br>tF(GAA)F/6:157916/157912,<br>tF(GAA)H1/8:237848/237986,<br>tF(GAA)H2/8:358478/358510,	tF(GAA)H2/8:358478/360000,
tQ(CUG)	1	1	0	tQ(CUG)M/13:808246/808340,	
tG(UCC)	4	2	1	tG(UCC)O/15:110962/109559,<br>tG(UCC)G/7:779687/779631,	tG(UCC)G/7:779687/778000,
tP(AGG)	2	2	0	tP(AGG)N/14:631846/631919,<br>tP(AGG)C/3:123577/123564,	
tS(GCU)	3	2	0	tS(GCU)O/15:274773/274755,<br>tS(GCU)F/6:191513/191585,	
tG(CCC)	2	2	0	tG(CCC)O/15:594354/592725,<br>tG(CCC)D/4:1257008/1257068,	
tC(GCA)	5	4	1	tC(GCA)P1/16:435893/435877,<br>tC(GCA)P2/16:775765/775808,<br>tC(GCA)B/2:643007/643075,<br>tC(GCA)G/7:707108/706939,	tC(GCA)P2/16:775765/777000,
tS(UGA)	4	3	1	tS(UGA)P/16:689646/689590,<br>tS(UGA)E/5:288443/288461,<br>tS(UGA)I/9:248931/249351,	tS(UGA)E/5:288443/288000,
tT(UGU)	5	4	1	tT(UGU)P/16:744355/744348,<br>tT(UGU)G1/7:661820/661761,<br>tT(UGU)G2/7:1004287/1004284,<br>tT(UGU)H/8:467061/466970,	tT(UGU)G1/7:661820/660000,
tW(UCA)	1	0	0		
tI(GAU)	1	0	0		

tT(UAG)	1	0	0		
tV(UAC)	3	2	1	tV(UAC)B/2:326792/326875, tV(UAC)D/4:488797/488806,	tV(UAC)B/2:326792/326000,
tS(CGA)	1	1	0	tS(CGA)C/3:228042/228871,	
tE(CUC)	2	2	1	tE(CUC)D/4:1017207/1017272, tE(CUC)I/9:197663/197748,	tE(CUC)D/4:1017207/1017000 ,
tV(CAC)	2	2	0	tV(CAC)D/4:1075472/1075481, tV(CAC)H/8:475706/475813,	
tL(GAG)	1	0	0		
**Total**	**299**	**256**	**47**		

Table S2: List of the yeast strains used in this study

Strain	Description	Genotype	Source	Use
YPB8	BY4742	MATα his3Δ1 leu2Δ0 lys2Δ0 ura3Δ0	Euroscarf	
YPB8-1	BRF1 FLAG	MATα his3Δ1 leu2Δ0 lys2Δ0 ura3Δ0 BRF1-3xFLAG::KanMX4	This study	
YPB8-2	TFC1 FLAG (DDY3927)	MATα his3Δ1 leu2Δ0 lys2Δ0 ura3Δ0 TFC1-3xFLAG::KanMX4	Suppl Ref (4)	
YPB8-5	TFC1 FLAG PAF1 HA	MATα his3Δ1 leu2Δ0 lys2Δ0 ura3Δ0 TFC1-3xFLAG::KanMX4, PAF1-6xHA::HphNT1	This study	Co-IP and ChIP
YPB8-10	BRF1 FLAG PAF1 HA	MATα his3Δ1 leu2Δ0 lys2Δ0 ura3Δ0 BRF1-3xFLAG::KanMX4, PAF1-6xHA::HphNT1	This study	Co-IP
yPB6	NZ16 (RPC128-FLAG)	MATa,ret1::HIS3, ura3,trp1, leu2, his3, met4, lys2, ade2, can1, cyhZ^R pNZ85: (parent PYE30 (CEN3)): TRP1, CEN3, ARS1, 6xHis-4xFLAG-RET1	George Kassavetis	
yPB6-9	RPC128-FLAG PAF1HA	MATa,ret1::HIS3, ura3,trp1, leu2, his3, met4, lys2, ade2, can1, cyhZ^R PAF1-6xHA::kanMX4 pNZ85: (parent PYE30 (CEN3)): TRP1, CEN3, ARS1, 6xHis-4xFLAG-RET1	This study	Co-IP
yPB7	NOY396	MATα ade2-1 ura3-1 trp1-1 leu2-3,112 his3-11,15 can1-100	Suppl Ref (5)	ChIP, Northern
yPB7-1	paf1Δ/DAS516	MATα ade2-1 ura3-1 trp1-1 leu2-3,112 his3-11,15 can1-100 paf1Δ::HIS3	Suppl Ref (5)	Northern
yPB7-2	rtf1Δ/DAS549	MATα ade2-1 ura3-1 trp1-1 leu2-3,112 his3-11,15 can1-100 rtf1Δ::HIS3	Suppl Ref (5)	Northern
yPB7-3	paf1Δ Rpc128myc	MATα ade2-1 ura3-1 trp1-1 leu2-3,112 his3-11,15 can1-100, paf1Δ::HIS3, RPC128-9xMyc::TRP	This study	ChIP, Northern

yPB7-4	rtf1Δ Paf1HA Rpc128myc	MATα ade2-1 ura3-1 trp1-1 leu2-3,112 his3-11,15 can1-100, rtf1Δ::HIS3 PAF1-6xHA::Hph, RPC128-9xMyc::TRP	This study	ChIP, Northern
yPB7-5	NOY396 DNApol2myc	MATα ade2-1 ura3-1 trp1-1 leu2-3,112 his3-11,15 can1-100 POL2-9xMyc::HphNT1	This study	ChIP
yPB7-6	paf1Δ DNAPol2-Myc	MATα ade2-1 ura3-1 trp1-1 leu2-3,112 his3-11,15 can1-100, paf1Δ::HIS3,POL2-9xMyc::HphNT1	This study	ChIP

Table S3A: Primers used for Real Time PCR estimations of ChIP samples

Gene/Amplicon	FORWARD	REVERSE
ARG1 (Pol II-transcribed)	AAAGGACATGTGGAAATTGATCGT	GGCCAAGAAAACATCCAAAG
PGD1 (Pol II-transcribed)	CGCAGGTCTACGCACAGCAGTC	GGTCAGCGGCGTGCCCATAA
tA(AGC)D	AGCAAGACGAGGTGGTCAAA	TGCGCTTTCAAAAATAACCA
tD(GUC)K	TTTTATTTTGCTTTTTATTCCTCCTGTA	CCATTTTGCTCCCGACTGC
tE(UUC)E1	GGCTGAATCCTGATAGTTTGAG	GGTAAAAACGATGGAGTGCAGAG
tG(CCC)O/SUF5	CGATAGTTGATTTTAATTCCAACAC	TCAATCAATTTGGGAAGACTCAG
tI(UAU)L	ATCCCCACCTCGAGCACTTTC	CCCGTATTGAATGACCCTTGAA
tL(CAA)A	AGTCCGCTTCCTACGGTTT	AAGAAAACGCTGATGCAGAA
tL(CAA)G1	CCGCGTTTGAAGCCGACTA	TCAAAGTATTAACCGGAAGAACACC
tN(GUU)L	ACTTAGCCACATTCGGGCTT	CGAGTTTCGGATGGGATCAA
tQ(CUG)M	ACAGATACAATCGCTCATCTCGA	AGGACTTGTCTGTCACACGC
tR(CCG)L/TRR4	TGTGATCCGGGTTCGAGT	CATCGTAGCCTCCGTTCTTT
tS(CGA)C/Sup61	CCTACGCCAAGCTGCTCTAC	GCCATAGTGCCATTTCGATT
tT(CGU)K/TRT2	CGGAGCCACAAATTTAGCAG	ATTTCAAATGCCCTCTGTGG
tY(GUA)O/Sup3	GCGGTTACGGTCTCAAGAAT	GGGAAGCATGAATTCTAACCA
tY(GUA)J2	CCCGGGAGATTTTTTTGTTT	AAAAGAGGCTACAAGAGTTCGTTAAT
SCR1 C	TGTAATGGCTTTCTGGTGGGATG	AGCTCTGCCCAGGACAAATTAC
SNR6 UP	GTCATCTTCCTGGACCTCATG	GCAATGAAACTCTAAAGTATCATCGATTCAG
SNR6 AB Box	GTTCCCCTGCATAAGGATGAACCG	GGAAGATAAAGATACACTGCTG
RPR1-Nuc A	CAGAAGGATCCCCACCTATG	CGACATTAACCCGGAGGAC
TelVIR	GCGTAACAAAGCCATAATGCCTCC	CTCGTTAGGATCACGTTCGAATCC

Table S3B: Primers used for Northern probing

Primer name/Target	Sequence
U4_82	GGTTTATAATTAAATTTCAACC
18S rRNA	GCTTATACTTAGACATGAAT
5S rRNA	GATTGCAGCACCTGAGTTTCGCGTTATGG
RT-Both/U6	TCTCTTTGTAAAACGGTTCATCCT
tE(UUC)E1	GGCTGAATCCTGATAGTTTGAG
tRNA Arg (UCU)	CACTCACGATGGGGGTCGAA
tRNA Leu (CAA)	GACCGCTCGGCCAAACAAC
tRNA iMet (CAU)	GTTTCGATCCGAGGACATCAG
tRNA Phe (GAA)	GCGCTCTCCCAACTGAGCT

Table S4: List of the Primers used for the tRNA-HySeq

Name	Sequence	Barcode
prMGL001	AATGATACGGCGACCACCGAGATCTACAC	
prMGL002	CAAGCAGAAGACGGCATACGAGATAGTCGTGTGACTGGAGTTCAGACGTGTGCTCTTCCG	ACGACT
prMGL003	CAAGCAGAAGACGGCATACGAGATACTGATGTGACTGGAGTTCAGACGTGTGCTCTTCCG	ATCAGT
prMGL004	CAAGCAGAAGACGGCATACGAGATATGCTGGTGACTGGAGTTCAGACGTGTGCTCTTCCG	CAGCAT
prMGL005	CAAGCAGAAGACGGCATACGAGATACGTCGGTGACTGGAGTTCAGACGTGTGCTCTTCCG	CGACGT
prMGL006	CAAGCAGAAGACGGCATACGAGATAGCTGCGTGACTGGAGTTCAGACGTGTGCTCTTCCG	GCAGCT
prMGL007	CAAGCAGAAGACGGCATACGAGATATCGTAGTGACTGGAGTTCAGACGTGTGCTCTTCCG	TACGAT
prMGL008	CAAGCAGAAGACGGCATACGAGATCGTCAGGTGACTGGAGTTCAGACGTGTGCTCTTCCG	CTGACG
prMGL009	CAAGCAGAAGACGGCATACGAGATCGTAGCGTGACTGGAGTTCAGACGTGTGCTCTTCCG	GCTACG
olMGL24	(Phos)AGATCGGAAGAGCGTCGTGTAGGGAAAGAGTGTAGATCTCGGTGGTCGC-(SpC18)-CACTCA-(SpC18)-TTCAGACGTGTGCTCTTCCGATCTATTGATGGTGCCTACAG-3	

III Supplementary References

1. Afgan, E. et al. The Galaxy platform for accessible, reproducible and collaborative biomedical analyses: 2016 Update. *Nucleic Acids Res*. **44** (WI), W3–W10 (2016).

2. Anders, S., Huber, W. Differential expression analysis for sequence count data. *Genome Biol* **11**: R106 (2010).

3. Ramírez, F., Dündar, F., Diehl, S., Grüning, B.A., Manke, T. deepTools: a flexible platform for exploring deep-sequencing data. *Nucl. Acids Res.* **42** (Web Server issue):W187-91. doi: 10.1093/nar/ gku365 (2014).

4. Simms, T. A. et al. TFIIIC binding sites function as both heterochromatin barriers and chromatin insulators in Saccharomyces cerevisiae. *Eukaryot. Cell* **7,** 2078–2086 (2008).

5. Zhang, Y., Smith, IV A. D., Renfrow, M. B. and Schneider, D. A. RNA Polymerase-associated factor 1 Complex (Paf1C) directly increases the elongation rate of RNA polymerase I and is required for efficient regulation of rRNA synthesis. *J. Biol. Chem.* **285,** 14152-14159 (2010).

IV Supplementary Figure Legends

Figure S1. Gene-specific differences in Paf1 occupancy on tRNA genes. Screen shots of Paf1 occupancy on tRNA genes located on different chromosomes viewed with Integrative Genomics Viewer. Screen shots of a window of >2 kb for seven genes are shown.

Figure S2. PAF1 complex is found at low levels on the pol III-transcribed genes. (A) The average occupancies at the pol III-transcribed gene loci (log_2 normalized tag counts) of Paf1, Leo1 and Cdc73 (30) on the 1kb upstream and downstream of TSS are plotted along with our previously published pol III occupancy data (40). The green bar marks the tRNA gene region on the X-axis. (B) Mean occupancy levels of the Ctr9, Paf1 and Rtf1 on the individual genes (33) within a window of -205 to 155 bp show a positive correlation. (C) Heat maps of the Rtf1 occupancies (34) within 1 kb upstream and downstream of TSS (bent arrow) on all the

tRNA genes (brown block) shows gene-specific variations in Rtf1 enrichment. Color gradient code is shown. (D) Average occupancy profiles (log_2 normalized Tag counts) of Rtf1 (34) are shown before (all tRNA) and after (pol II out) filtering out the tRNA genes having pol II-transcribed ORFs within 300 bp upstream or downstream of their ends. (E) Schematic representation of Real Time PCR amplicon positions for *SCR1*, *RPR1* and *SNR6* genes. The shaded rectangles demarcate the mature transcript.

Figure S3. RNA estimations by northern blotting. Transcript levels were estimated in the wild type [W], *paf1Δ* [P] and *rtf1*Δ [R] cells. Full-length northern blots used for probing with gene-specific primers are shown. (A) Left panel shows a typical membrane after transfer but before probing. Exposure of the membrane to UV reveals different RNA bands as visible in GelDoc. (B) The three images represent the full-length northern blots for the respective cropped images shown in the Fig. 3. (C) Heat map of the normalized levels of mature tRNAs in the wild type and *paf1Δ* cells from tRNA-HySeq data is shown. Individual tRNA reads were merged into 70 groups according to sequence identities (marked on the right hand side) and a comparison of the log_2 transformed, normalized read counts of each group (color code given at the bottom) is shown. (D) Rtf1 deletion does not cause change in individual tRNA levels. The log_2 transformed, normalized read counts of each tRNA from the wild type and *rtf1*Δ cells obtained in the Hyseq data are compared.(E) Pol III occupancy on the genomic DNA (40) and pol III levels bound to the nascent transcript (CRAC) levels (53) of the tRNA genes do not show a correlation.

Figure S4. Yeast γ-H2A (Phosphorylated H2A S129) levels without or with genotoxins exposure. Measurements were made by performing the ChIP and Real Time PCR. Dots denote insignificant changes, while rest were significant with highest p values <0.05 (A) Very low level of γ-H2A is found on pol III-transcribed genes in the normally cycling wild type cells. (B) In *paf1Δ* cells, the γ-H2A levels are higher than in the wild type cells, even without an exposure to any genotoxin. Dots denote insignificant changes (p value between 0.06-0.09), while rest were significant changes with p values <0.05. (C) Fold increase in γ-H2A levels when both *paf1Δ* and the wild type cells are treated with genotoxins for 2hr. Ratios *paf1Δ*/wild type were calculated by using the data in the Fig. 3A for HU and Fig. 3B for MMS treated conditions. (D) As compared with the wild type levels, the DNA pol2 levels in the *paf1Δ* cells change in a gene-specific manner, without any genotoxin treatment.

Figure S5. Genotoxin MMS effect on pol III occupancy is independent of Paf1. (A) Pol III occupancy in the MMS-treated wild type cells are lower than the untreated levels; all p values <0.01. (B) Pol III occupancies in the *paf1Δ* cells decrease significantly (p<0.04) upon exposure to MMS on most of the genes. The genes marked with dots show insignificant changes with p values 0.062 (*tL(CAA)A*) and 0.055 (*tY(GUA)J2*). (C) Exposure to MMS results in similar decrease of pol III in the wild type and *paf1Δ* cells. The y axis shows the ratio of pol III occupancies in MMS treated/untreated cells calculated by using the data in the panel A for the wild type and panel B for the *paf1Δ* cells.

Figure S6. Heat maps of the enrichment of tRNAs and Paf1 around the TAD boundaries and replication origins are shown. The color grade on the heatmaps represent the normalized enrichment in each genomic bin. (A) Enrichment of tRNA start sites .The columns in the heatmaps represent the genomic bins of 1kb centered around the replication origins and the TAD boundaries. Rows in the heatmaps represent different replication origins and the TAD boundaries. (B) Paf1 enrichment (ChIP-exo signal) are shown. The columns in the heatmaps represent the genomic bins of 100bp centered around the replication origins and the TAD boundaries. Rows in the heatmaps represent different replication origins and the TAD boundaries.

Figure S1

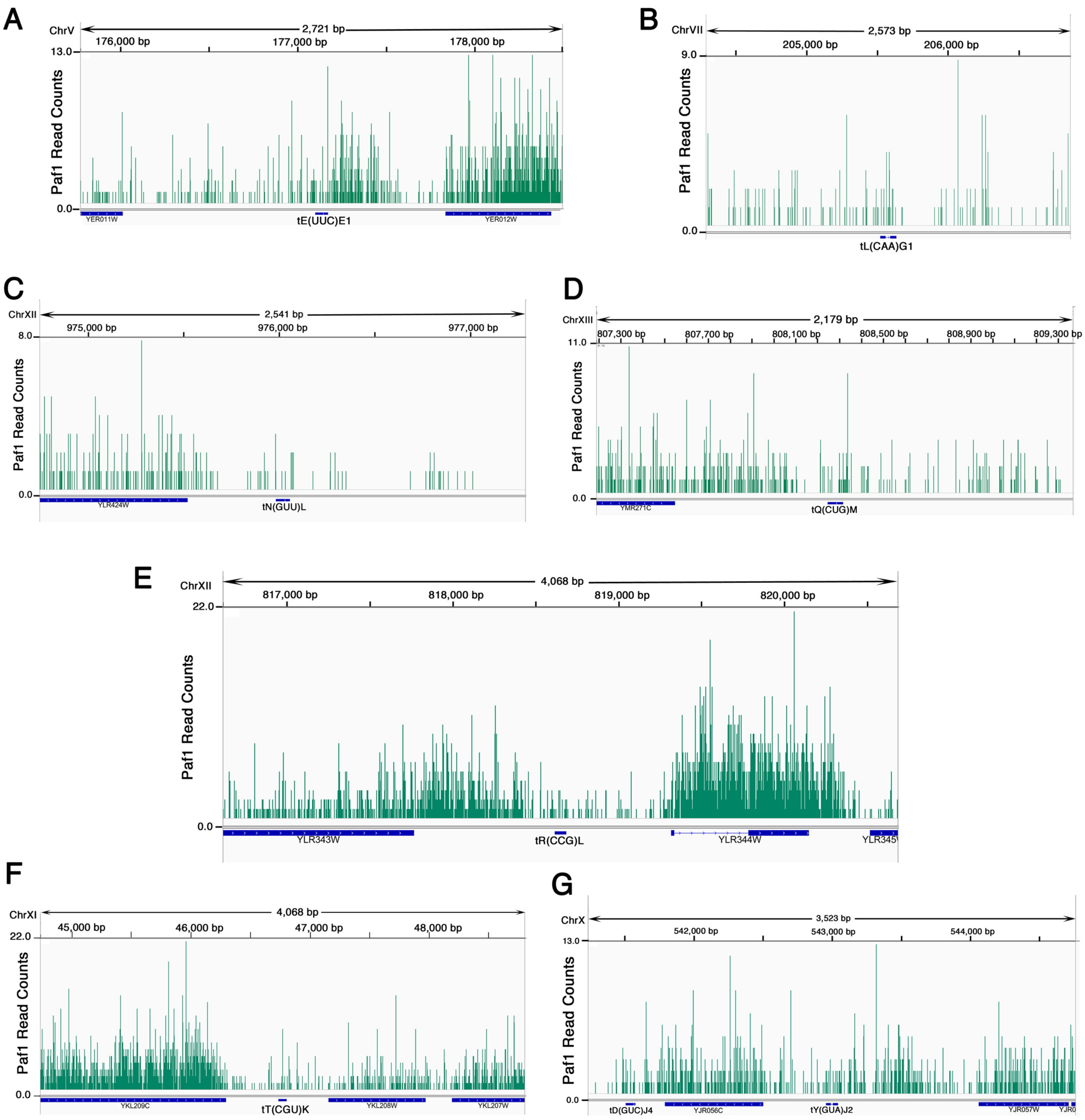

A
ChrV
2,721 bp
176,000 bp
177,000 bp
178,000 bp
13.0
0.0
Paf1 Read Counts
YER011W
tE(UUC)E1
YER012W
B
ChrVII
2,573 bp
205,000 bp
206,000 bp
9.0
0.0
Paf1 Read Counts
tL(CAA)G1
C
ChrXII
2,541 bp
975,000 bp
976,000 bp
977,000 bp
8.0
0.0
Paf1 Read Counts
YLR424W
tN(GUU)L
D
ChrXIII
2,179 bp
807,300 bp
807,700 bp
808,100 bp
808,500 bp
808,900 bp
809,300 bp
11.0
0.0
Paf1 Read Counts
YMR271C
tQ(CUG)M
E
ChrXII
4,068 bp
817,000 bp
818,000 bp
819,000 bp
820,000 bp
22.0
0.0
Paf1 Read Counts
YLR343W
tR(CCG)L
YLR344W
YLR345
F
ChrXI
4,068 bp
45,000 bp
46,000 bp
47,000 bp
48,000 bp
22.0
0.0
Paf1 Read Counts
YKL209C
tT(CGU)K
YKL208W
YKL207W
G
ChrX
3,523 bp
542,000 bp
543,000 bp
544,000 bp
13.0
0.0
Paf1 Read Counts
tD(GUC)J4
YJR056C
tY(GUA)J2
YJR057W

Figure S2

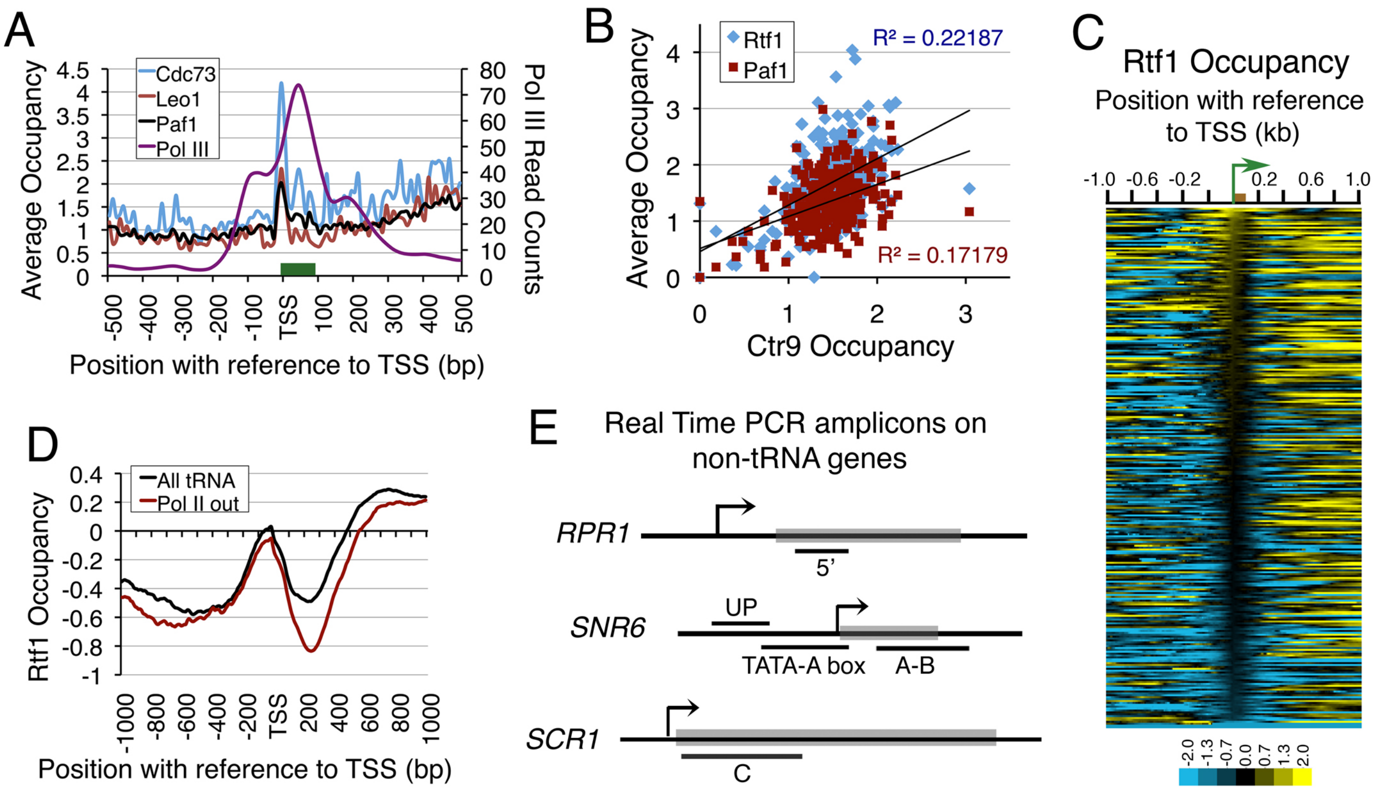

A
Average Occupancy
Pol III Read Counts
Cdc73
Leo1
Paf1
Pol III
Position with reference to TSS (bp)
B
Rtf1
Paf1
R² = 0.22187
R² = 0.17179
Average Occupancy
Ctr9 Occupancy
C
Rtf1 Occupancy
Position with reference to TSS (kb)
D
All tRNA
Pol II out
Rtf1 Occupancy
Position with reference to TSS (bp)
E
Real Time PCR amplicons on non-tRNA genes
RPR1
5'
SNR6
UP
TATA-A box
A-B
SCR1
C

Figure S3

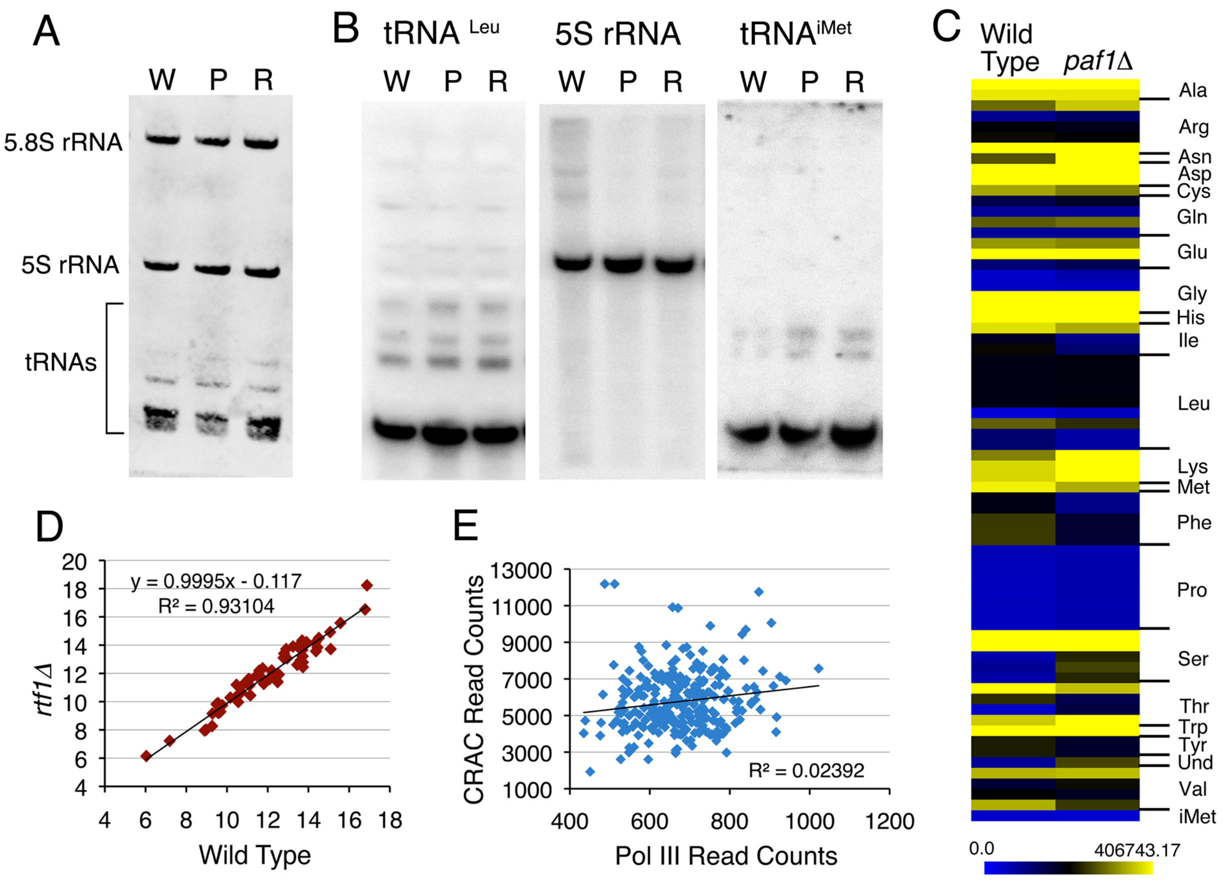

A
W P R
5.8S rRNA
5S rRNA
tRNAs
B
tRNA Leu
5S rRNA
tRNAiMet
W P R
W P R
W P R
C
Wild Type
paf1Δ
Ala
Arg
Asn
Asp
Cys
Gln
Glu
Gly
His
Ile
Leu
Lys
Met
Phe
Pro
Ser
Thr
Trp
Tyr
Und
Val
iMet
0.0
406743.17
D
y = 0.9995x - 0.117
R² = 0.93104
rtf1Δ
Wild Type
20
18
16
14
12
10
8
6
4
4 6 8 10 12 14 16 18
E
CRAC Read Counts
13000
11000
9000
7000
5000
3000
1000
R² = 0.02392
400 600 800 1000 1200
Pol III Read Counts

Figure S4

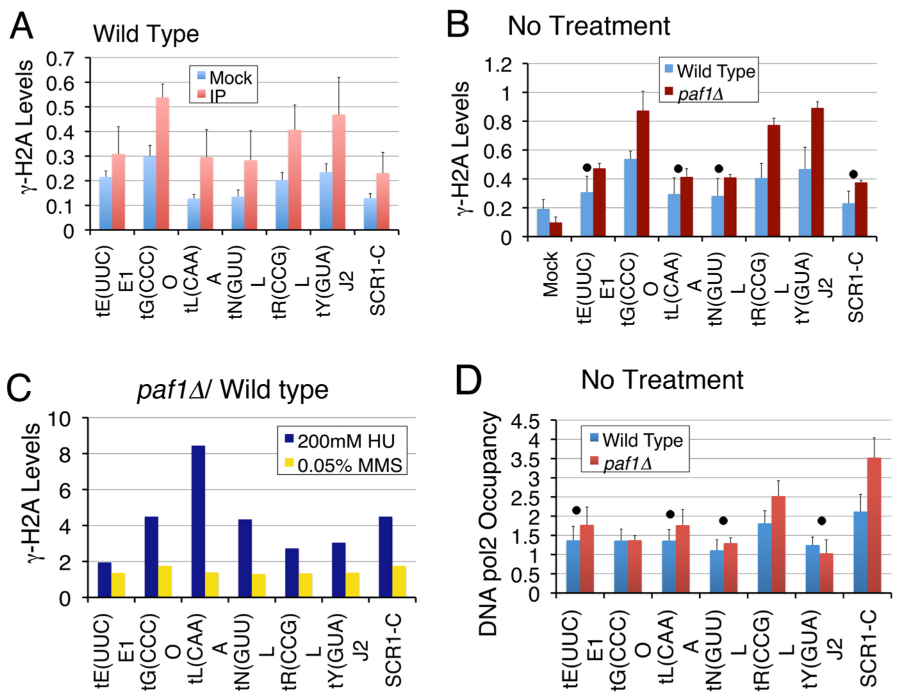
A
Wild Type
γ-H2A Levels
Mock
IP
tE(UUC)E1
tG(CCC)O
tL(CAA)A
tN(GUU)L
tR(CCG)L
tY(GUA)J2
SCR1-C
B
No Treatment
γ-H2A Levels
Wild Type
paf1Δ
Mock
C
paf1Δ/ Wild type
γ-H2A Levels
200mM HU
0.05% MMS
D
No Treatment
DNA pol2 Occupancy
Wild Type
paf1Δ

Figure S5

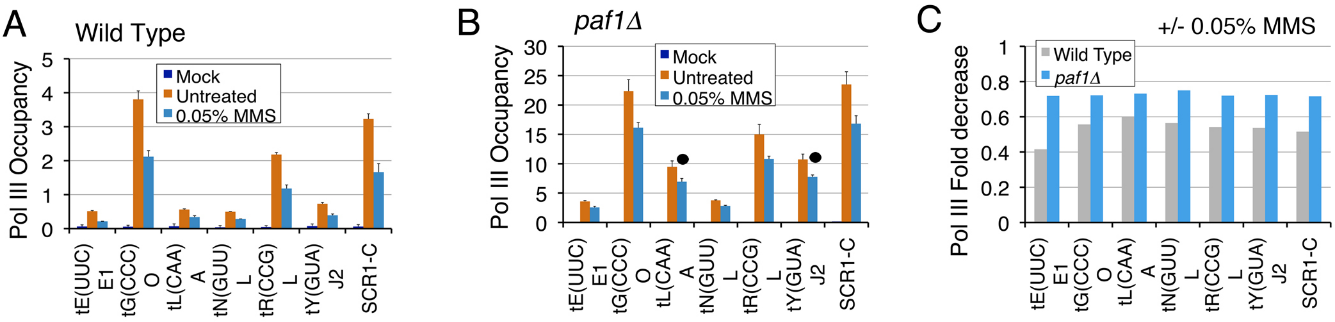

A
Wild Type
Pol III Occupancy
Mock
Untreated
0.05% MMS
B
paf1Δ
Pol III Occupancy
C
+/- 0.05% MMS
Pol III Fold decrease
Wild Type
paf1Δ
tE(UUC)E1
tG(CCC)O
tL(CAA)A
tN(GUU)L
tR(CCG)L
tY(GUA)J2
SCR1-C

Figure S6

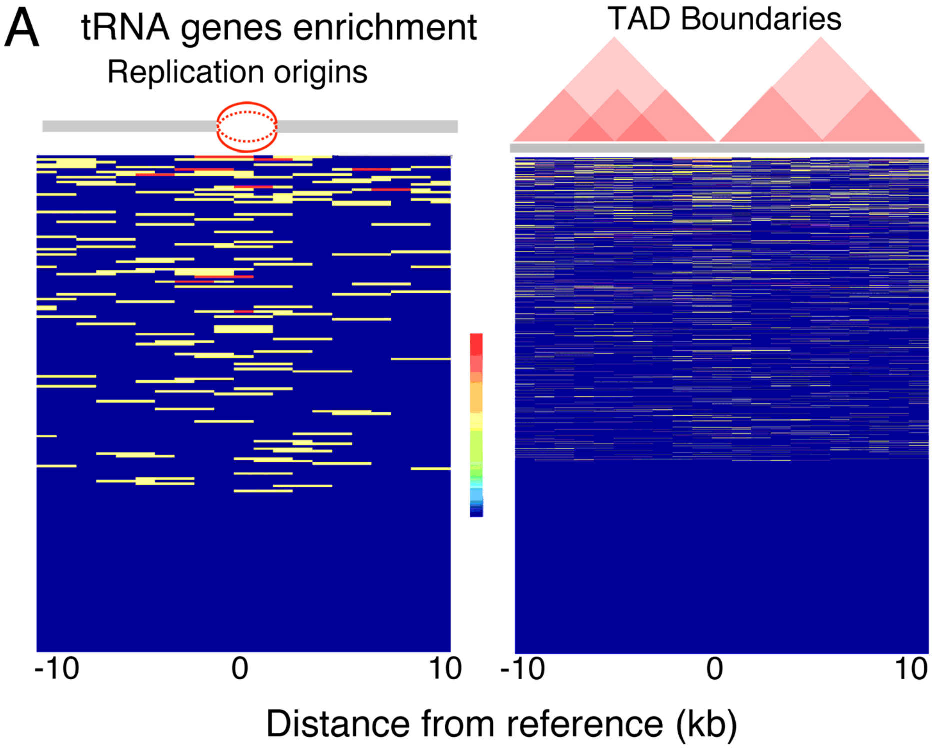
A
tRNA genes enrichment
Replication origins
TAD Boundaries
-10
0
10
-10
0
10
Distance from reference (kb)

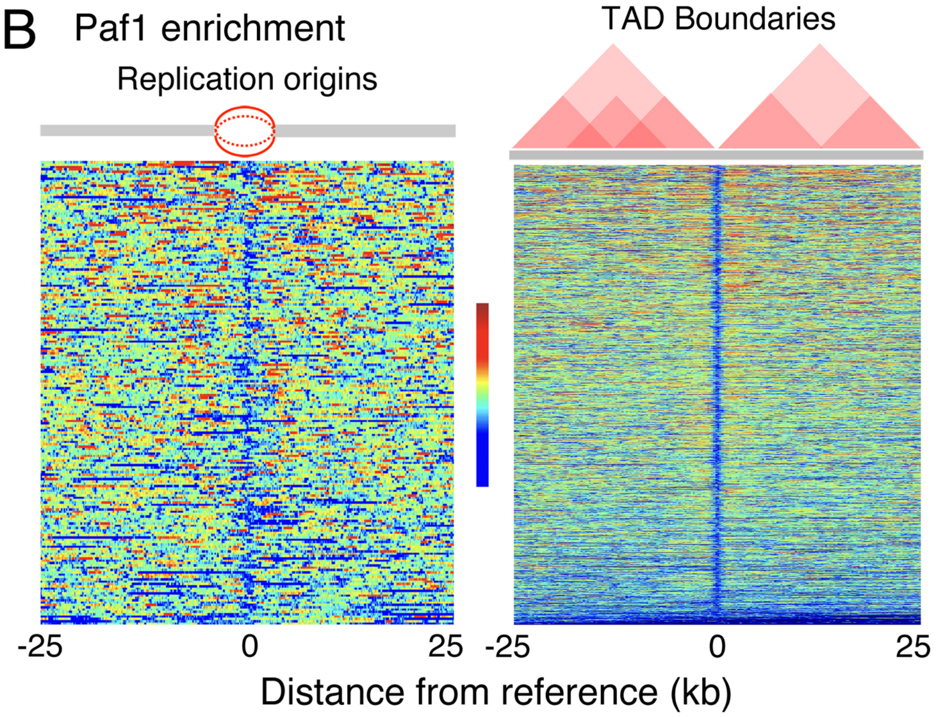
B
Paf1 enrichment
Replication origins
TAD Boundaries
-25
0
25
-25
0
25
Distance from reference (kb)
